# PanDrugs: a novel method to prioritize anticancer drug treatments according to individual genomic data

**DOI:** 10.1186/s13073-018-0546-1

**Published:** 2018-05-31

**Authors:** Elena Piñeiro-Yáñez, Miguel Reboiro-Jato, Gonzalo Gómez-López, Javier Perales-Patón, Kevin Troulé, José Manuel Rodríguez, Héctor Tejero, Takeshi Shimamura, Pedro Pablo López-Casas, Julián Carretero, Alfonso Valencia, Manuel Hidalgo, Daniel Glez-Peña, Fátima Al-Shahrour

**Affiliations:** 1 Spanish National Cancer Research Centre (CNIO), 3rd Melchor Fernandez Almagro st., E-28029 Madrid, Spain; 2 Computer Science Department - University of Vigo, Vigo, Spain; 3 Biomedical Research Centre (CINBIO), Vigo, Spain; 4 Spanish National Bioinformatics Institute (INB), Madrid, Spain; 5 Department of Physiology - University of Valencia, Valencia, Spain; 6 Loyola University Chicago Stritch School of Medicine, Maywood, IL USA; 7 Beth Israel Deaconess Medical Center, Boston, USA

**Keywords:** Precision oncology, Personalized medicine, Translational bioinformatics, Cancer genomics, In silico prescription, Targeted therapy, Druggable genome

## Abstract

**Background:**

Large-sequencing cancer genome projects have shown that tumors have thousands of molecular alterations and their frequency is highly heterogeneous. In such scenarios, physicians and oncologists routinely face lists of cancer genomic alterations where only a minority of them are relevant biomarkers to drive clinical decision-making. For this reason, the medical community agrees on the urgent need of methodologies to establish the relevance of tumor alterations, assisting in genomic profile interpretation, and, more importantly, to prioritize those that could be clinically actionable for cancer therapy.

**Results:**

We present PanDrugs, a new computational methodology to guide the selection of personalized treatments in cancer patients using the variant lists provided by genome-wide sequencing analyses. PanDrugs offers the largest database of drug-target associations available from well-known targeted therapies to preclinical drugs. Scoring data-driven gene cancer relevance and drug feasibility PanDrugs interprets genomic alterations and provides a prioritized evidence-based list of anticancer therapies. Our tool represents the first drug prescription strategy applying a rational based on pathway context, multi-gene markers impact and information provided by functional experiments. Our approach has been systematically applied to TCGA patients and successfully validated in a cancer case study with a xenograft mouse model demonstrating its utility.

**Conclusions:**

PanDrugs is a feasible method to identify potentially druggable molecular alterations and prioritize drugs to facilitate the interpretation of genomic landscape and clinical decision-making in cancer patients. Our approach expands the search of druggable genomic alterations from the concept of cancer driver genes to the druggable pathway context extending anticancer therapeutic options beyond already known cancer genes. The methodology is public and easily integratable with custom pipelines through its programmatic API or its docker image. The PanDrugs webtool is freely accessible at http://www.pandrugs.org.

**Electronic supplementary material:**

The online version of this article (10.1186/s13073-018-0546-1) contains supplementary material, which is available to authorized users.

## Background

Identifying the most appropriate therapies from cancer genome data is a major challenge in personalized cancer medicine. Standard of care treatments are commonly selected following criteria such as: cancer type; stage; patient status; and/or the presence of prognostic and predictive biomarkers. However, cancer treatment could be revolutionized if the information contained in large genomic datasets were to be systematically deconvoluted in terms of potential treatments [1]. Here, the identification and evaluation of somatic alterations and their collective impact on tumor progression pose considerable challenges to their clinical application [2, 3]. More specifically, physicians and researchers are challenged with long lists of tumor-specific genomic variants where most variants are either clinically “unactionable,” their biological role unknown, or they are irrelevant for tumor biology [4]. In addition, the current list of cancer driver genes [5] has clinical limitations since genomic alterations in these genes may be essential for oncogenesis, tumor cell growth, and survival; but the same genes may not be targetable by current therapies [6]. Moreover, very recent studies have revealed that cancer gene lists are still incomplete and that there are many more cancer genes yet to be discovered [7–9]. In this scenario, it is essential to develop new methodologies to analyze genetic alterations in terms of treatment options, helping to prioritize those that could be useful for the management of cancer patients.

Several remarkable efforts have addressed the prioritization of genomic alterations [10–13]. These methods exploit the extensive literature and knowledge available in public repositories to catalogue cancer genomic variants and their impact on biological functions, although none of these methodologies directly link genomic alterations to potential therapies. Tools such DGIdb [14], OncoKB [15], and the Cancer Genome Interpreter (CGI) [16] have been developed to identify clinically actionable genomic alterations in tumors. Although these tools demonstrate the potential of targeted therapies and provide drug repurposing strategies, they present some limitations. They only consider known cancer driver genes for drug prescription, they are based exclusively on somatic DNA alterations, the therapeutic options are restricted to “one target - one drug” ignoring multiple targetable mutations and the protein pathway-specific activity, and they do not provide a prioritized list of treatments based on clinical, biological, and pharmacological evidence.

Here we introduce PanDrugs, a new computational resource to propose drug therapies from genome-wide experimental results, including variant and gene lists. PanDrugs expands cancer therapeutic options by taking into account multiple genomic events potentially responsive to a treatment, the pathway context [17], and the pharmacological evidence reported in large-scale experiments [18, 19].

## Implementation

### PanDrugs database

**Fig. 1:**
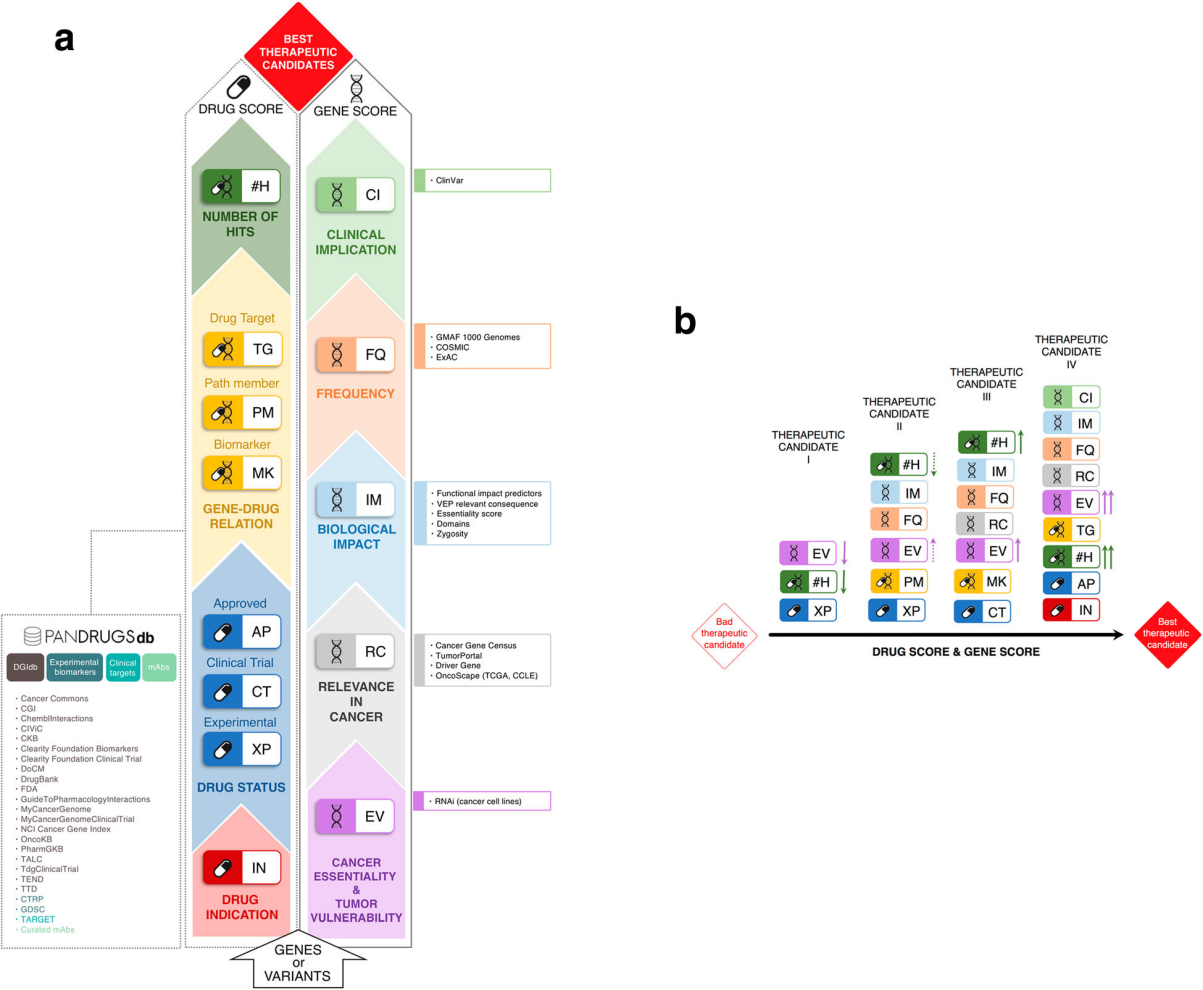
PanDrugs score calculation. **a** Overview of the DScore and GScore calculation and their corresponding annotation sources. PanDrugs considers drug indication and status, gene–drug associations and number of hits to calculate the DScore. GScore is estimated according to gene essentiality and tumoral vulnerability, gene relevance in cancer, the biological impact of mutations, the frequency of gene alterations, and their clinical implications. **b** PanDrugs considers the “Best therapeutic candidates” based on the accumulated and weighted scoring of GScore and DScore

The PanDrugs database (PanDrugsdb) has been implemented to store gene–drug relationships. PanDrugs methodology mines PanDrugsdb to provide a catalogue of prioritized candidate drugs and targetable genes estimated from a list of variants (or genes) provided by a user (Fig. 1a).

Pharmacological data and drug annotations available in PanDrugsdb were collected from 24 databases. These included 18 sources with information curated by experts and gene–drug associations obtained from experimental drug screenings: The Cancer Therapeutics Response Portal [19] and GDSC [20] (Additional file 1: Table S1). Since different sources employ a variety of non-standardized identifiers to mention the same compound, drug names were standardized in order to be consistently integrated within PanDrugsdb (Additional file 1: Materials and Methods). Following this, drug annotations were enriched with additional information regarding drug families, drug indication status, cancer type, and therapy description (Additional file 1: Figure S1 and S2). Gene–drug relationships were also annotated with the type of gene–drug relation (i.e. drug target or biomarker), drug sensitivity or resistance response, and the type of genomic alteration associated to the drug response.

The current version of PanDrugsdb includes 9092 drugs, 4804 unique genes, and 43,909 direct and non-redundant gene–drug interactions. The database is built in MySQL RDBMS. Full details regarding PanDrugsdb implementations and PanDrugs software are described in Additional file 1: Materials and Methods.

### PanDrugs nomenclature

PanDrugs categorizes druggable genes as: (1) direct targets; (2) biomarkers; and (3) pathway members.

The term “direct targets” includes those genes that contribute to disease phenotype and can be directly targeted by a drug (i.e. small molecule or monoclonal antibody). For instance, *BRAF* is a direct target for vemurafenib [21]. “Biomarkers” refers to genes that have a genetic status associated with drug response (according to clinical or pre-clinical evidence) but the protein product is not the drug target itself. For example, *BRCA*-mutated cancers that respond to poly-ADP-ribose polymerase (*PARP*) inhibitors [22], *PTEN* loss that is associated with decreased sensitivity of colorectal cancer tumors to anti-EGFR antibodies [23], or mutations in *TSC1/2* as clinically approved biomarkers of PI3K/Akt/mTOR inhibitor response [24, 25]. PanDrugs “biomarkers” information was obtained from manually curated databases (see Additional file 1: Materials and Methods for details) and from experimental assays in cancer cell lines (GDSC and CTRP).

Targeted therapies may target cell signals that are needed for cancer cells to develop, proliferate, and invade. Drugs targeting the activity of the surrounding interactors in the biological pathway of a mutated gene could: (1) produce the same downstream effect as targeting the mutated gene itself; (2) enhance response by synergistic effects; and (3) be used in combination to avoid possible compensatory drug resistance mechanisms [26–29]. Following this paradigm, PanDrugs includes “pathway member” referring to all those downstream druggable targets taking advantage of the pathway background underlying the user’s gene list. Interestingly, this paradigm unlocks alternative therapeutic ways for untargetable genes.

Finally, PanDrugs analyzes the “collective gene impact” defined as the number of druggable genes (direct targets, biomarkers, and pathway members) in the input list that points to a particular drug. Those drugs capable of targeting the largest number of druggable genes are prioritized.

### PanDrugs uses two scores to prioritize cancer treatments

PanDrugs calculates two scores integrating a variety of clinical, biological, and pharmacological sources and databases to suggest tailored anticancer therapies based on user supplied variant lists and PanDrugsdb (Fig. 1a). Gene Score (GScore) is in the range of 0–1 based on the level of evidence supporting gene clinical implication and its biological relevance in cancer (Additional file 1: Figure S3A). Drug Score (DScore) estimates drug response and treatment suitability (Additional file 1: Figure S3B). A larger number of supporting databases, curated annotation, and clinical impact enhance the weight in both GScore and DScore calculation. Full descriptions of GScore and DScore calculations are available in Additional file 1: Materials and Methods.

GScore has been implemented to consider: (1) genomic feature evidence by mutation consequence, functional impact, and population allele frequency; (2) relevance in cancer estimated by Cancer Gene Census (CGC) of COSMIC v84 [30], TumorPortal resource [31], Tamborero et al. [32], and OncoScape [33]; (3) essentiality from RNA interference (RNAi) experiments in cancer cell lines from Achilles project [34, 35] and; (4) clinical implications based on its pathogenicity supporting evidence (taken from COSMIC and ClinVar). GScore weight assignation for non-ranked gene lists and for VCF files are described in Additional file 1: Tables S2 and S3, respectively.

DScore is calculated using PanDrugsdb to evaluate the therapeutic implications of those altered genes previously employed for GScore calculation. DScore takes into account: (1) drug-cancer type indication (from the FDA and clinicaltrials.gov); (2) drug clinical status (approved by the FDA, clinical trials, or preclinical); (3) gene–drug relationship (i.e. direct target, biomarker, or pathway member); (4) number of curated databases supporting that relationship (i.e. database factor); and (5) collective gene impact (Additional file 1: Figure S3C). DScore has values from − 1 to 1 where negative values correspond to drug unresponsiveness and positive values to drug sensitivity (Additional file 1: Figure S3B).

PanDrugs provides a prioritized list of candidate drugs considering GScore and DScore values. Those drug therapies supported by scores nearest to 1 in both GScore and DScore will have more evidence for their effectiveness in cancer treatment and will be considered “Best therapeutic candidates” by PanDrugs (Fig. 1b).

### Exploiting pathway information to increase therapeutic options

**Fig. 2:**
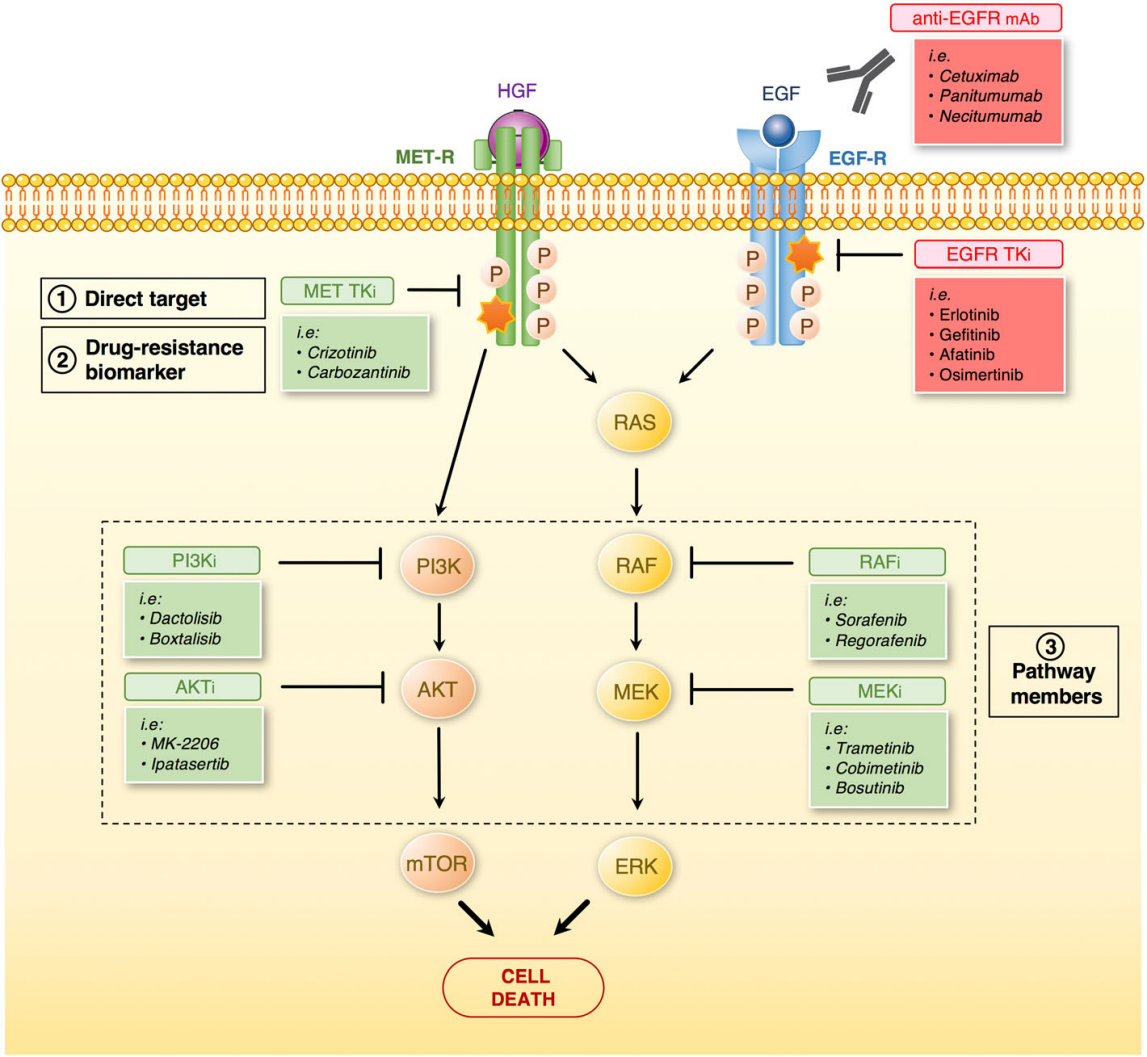
Possible scenarios for PanDrugs therapeutic candidates. PanDrugs proposes three potential types of druggable candidates. This includes: (1) direct targets, a gene that contributes to a disease phenotype and can be directly targeted by a drug; (2) drug-resistance biomarkers, a gene which genetic status is associated with a drug response from clinical or pre-clinical evidence but its protein product is not the direct target of the drug; and (3) pathway members, a targetable gene located downstream to the altered one. To illustrate this, tumors mutated in EGFR carrying MET amplifications will not respond to EGFR inhibitors (*red*). PanDrugs proposes as therapeutic strategy MET inhibitors and targeting MET downstream proteins (*green*) to drive tumor cell death

PanDrugs expands the anticancer therapeutic arsenal suggesting drugs to target genes located downstream to the altered gene(s). To this end, PanDrugs integrates previously modelled biological circuit information (e.g. signaling pathways) [36], the interaction types between nodes (activation or inhibition), and the gene functional role (oncogene or tumor suppressor). Ideally, a perfect gene–drug(s) solution will meet the following criteria: (1) the gene is affected by activating point mutations (predicted by functional impact algorithms or confirmed by databases/literature); (2) the gene is essential in synthetic lethal screenings; (3) the gene is sensitive to specific drugs included in PanDrugs; and (4) an FDA drug is approved or under clinical trial that targets the gene. Although it is known that only few genomic alterations will follow these stringent criteria, the emerging “druggable genome” concept opens the whole genome to therapeutic intervention. In other words, both a given mutated gene and its interactions are putative drug targets [37, 38]. Following this paradigm, PanDrugs offers a systems biology framework to propose drugs that arise as rational therapeutic candidates. For example, *MET* amplification plays a role in acquired resistance to EGFR inhibitors of patients with *EGFR*-mutated tumors by activating MAPK and PI3K/AKT signaling pathways [39, 40]. Combination therapy of EGFR and MET inhibitors is used to block both MET and EGFR signaling pathways [41]. In this scenario, PanDrugs proposes the following as therapeutic options: (1) avoiding EGFR inhibitors alone due to the known lack of sensitivity; (2) the usage of MET inhibitors that can overcome resistance of *EGFR*-TKIs; and (3) targeting downstream druggable genes (i.e. *RAF*, *MEK*) with available drugs (i.e. Sorafenib, Trametinib) blocking the oncogenic consequences of the pathway overstimulation [42, 43] (Fig. 2).

### PanDrugs web tool and application programming interface

PanDrugs is available as a user-friendly web tool with preloaded queries and demo examples at http://www.pandrugs.org. The detailed user manual is accessible online at https://www.pandrugs.org/#!/help. The server supports three alternative types of input: (1) single and multiple queries (gene lists); (2) standard VCF files; and (3) a ranked list of genes, where ranking is obtained from experimental observations (i.e. RNA-sequencing experiments). Regular analysis of a 500-line VCF file takes an average time of ~ 6 min in the PanDrugs server. The results page provides a panel with basic statistics of the query, pie-charts depicting clinical status distribution and families for the drugs proposed, and a bubble plot representing GScore and DScore together with the best candidate therapies suggested by PanDrugs. Moreover, the tool generates a ranked summary table of the treatments with raw scores and links to external databases (Additional file 1: Figure S4). In addition to GScore and DScore, the summary table displays comprehensive and sortable information about genes, drugs, type of gene–drug interaction, drug family, drug clinical status, type of therapy available (only for the approved drugs), and sources of annotation employed. PanDrugs uses KEGG pathways to map the relationship between input genes and pathway members suggested as candidate drug targets. Our tool also supports drug queries to explore the gene–drug interactions available in PanDrugsdb providing the subsequent summary table. All results generated by PanDrugs are easily downloadable in standard formats (i.e. CSV, PNG, PDF, etc.). PanDrugs provides a REST-based Application Programming Interface (API) allowing developers to make queries directly to PanDrugsdb, to incorporate output from PanDrugs within their own algorithms, and to combine the tool as a novel module in NGS analysis pipelines integrating genetic data and therapeutic alternatives. PanDrugs is also available as a docker image at https://github.com/sing-group/pandrugs-docker.

## Results

### PanDrugs in The Cancer Genome Atlas (TCGA) data

PanDrugs has been systematically applied to a cohort of 7069 samples from the TCGA project that correspond to 20 different tumor types (Additional file 1: Figure S5). File sources employed for the TCGA analysis are listed in Additional file 1: Table S4. Databases used in the study and their corresponding versions are detailed in Additional file 1: Table S5. Full results may be interactively accessed at the PanDrugs website (http://www.pandrugs.org/).

The PanDrugs analysis of TCGA tumors showed that the GScore distribution of genes affected by genomic alteration events (SNVs + indels + Copy Number Variations (CNVs)) drops drastically at GScore = 0.4. In agreement with previous studies, this observation suggests that most genomic alterations in TCGA patients have little evidence of being associated with cancer and, in consequence, are poorly annotated in public databases [31, 44] (Additional file 1: Figure S6A). Genes with a GScore > 0.4 and carrying at least one mutational and/or CNV event were used to identify potential therapies and were found present in > 6000 of the 7069 TCGA samples (Additional file 1: Figure S6B, S6C; Table S6). We decided to use this threshold for TCGA analysis to establish a compromise solution between gene annotation quality and retaining the largest number of patients. For instance, *EGFR* mutations across different TCGA tumor types exhibit a 0.23 < GScore < 0.82 while mutations in *KRAS* have GScore values in the range of 0.36–0.97. These GScores underline the biological relevance and clinical utility of both *KRAS* and *EGFR* genomic alterations in cancer. Differences in GScore values reflect the higher frequency of *KRAS* hotspot mutations and their well-known pathogenicity in contrast to the *EGFR* mutational heterogeneity and its broader functional impact and pathological diversity (Additional file 1: Figure S7). In particular, *EGFR*_GScore_ ~ 0.8 reveals those well-known mutations with clinical significance related to drug response (L858R) while *EGFR*_GScore_ ~ 0.4 corresponds to mutations residues that are not located in the protein kinase domain and that are less frequently found in cancer patients but that may have a deleterious functional effect (i.e. *EGFR* p.L62R). *KRAS*_GScore_ ~ 0.9 represents well-described and very frequent driver mutations located in exon 2 codons 12 and 13 with clinical significance as diagnostic, prognostic, and predictive biomarkers.

Using a GScore threshold our observations showed that an in silico prescription of approved drugs for direct targets plus biomarkers offered treatments for 65% of patients when point mutations, indels, and CNVs are considered simultaneously. (Additional file 1: Figure S8A). Notably, PanDrugs is able to extend drug prescription for 86% of TCGA patients by exploiting pathway member–drug associations (Additional file 1: Figure S8B).

The PanDrugs TCGA analysis was also used to evaluate the potential of non-driver genes as effective targets for cancer treatment by selecting the most frequently altered genes in TCGA patients annotated in PanDrugsdb. This selection was carried out for every TCGA tumor type by considering the top five genes according to frequency in small variants (point mutations and indels) and CNVs separately. Following these criteria, we obtained 200 alterations located in 100 genes (Additional file 1: Table S7). Interestingly, 54% of these most frequently altered genes were found druggable but are currently labelled as non-driver genes [45, 32]. Our results strongly suggest that the extension of genomic events detection beyond the known cancer driver genes can help in finding additional effective therapies for cancer treatment.

We compared PanDrugs’ performance by applying our methodology to the TCGA cohort previously used in other studies [46, 47] This comparative TCGA analysis showed that the PanDrugs pathway member approach expands therapeutic options for FDA-approved drugs to an average percentage of 93.41% in TCGA patients (Additional file 1: Figure S9). This result shows that the PanDrugs pathway member strategy might be useful to complement current in silico prescription tools.

### Comparison with other tools

**Fig. 3:**
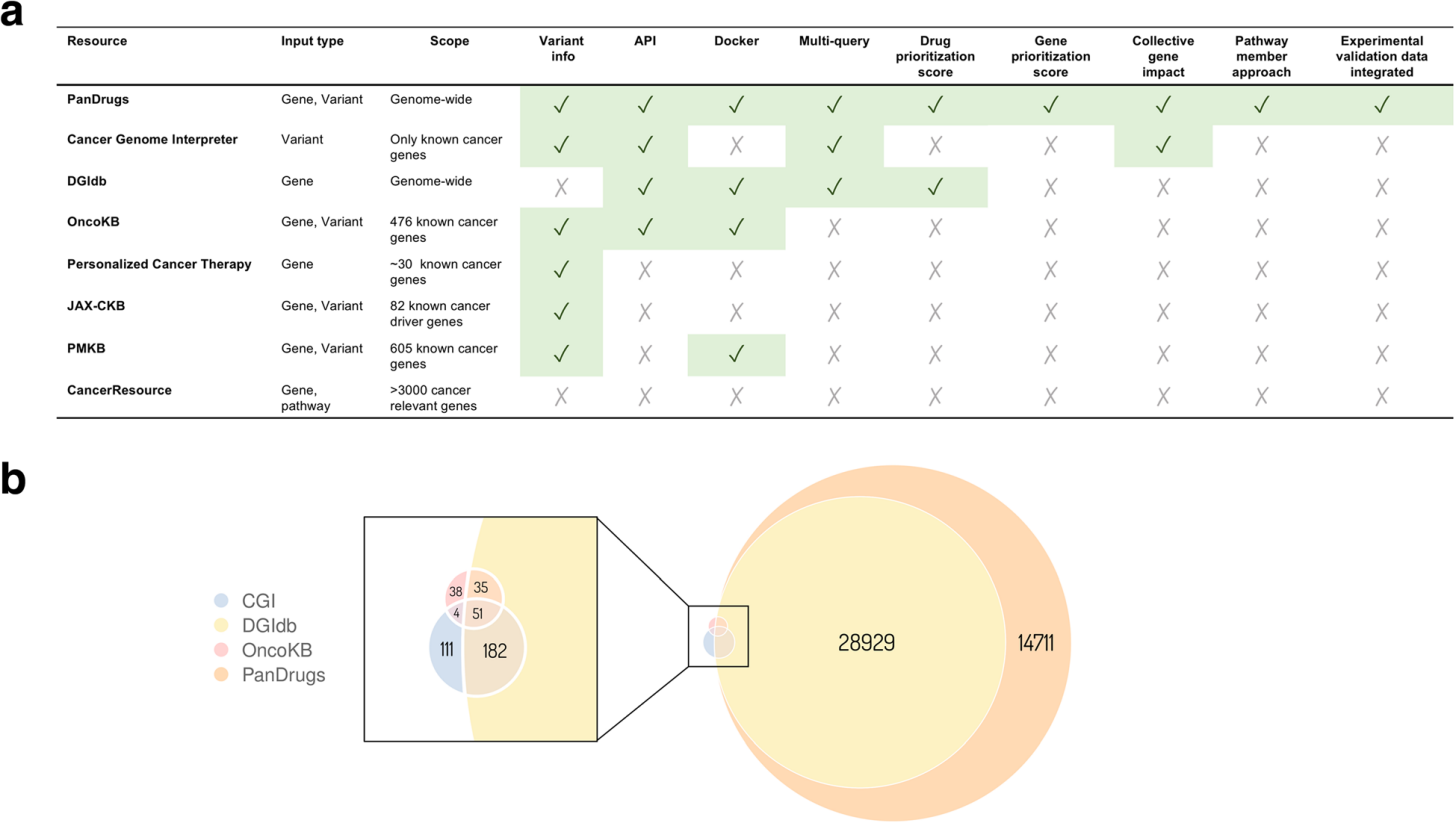
**a** Comparison of current in silico drug prescription tools based on genomic data. **b** *Venn diagram* for drug–gene associations available in DGIdb v3.0.2, Cancer Genome Interpreter, OncoKB, and PanDrugs. Global data for associations from CancerResource and Personalized Cancer Therapy is not accessible. Total numbers for non-redundant drug–gene interactions after drug standardization using PubChem to compare the resources are 29,197 (DGidb), 349 (CGI), 129 (OncoKB), and 43,909 (PanDrugs)

We compared PanDrugs’ performance to DGIdb, OncoKB, CGI, CancerResource (CR) [48], Personalized Cancer Therapy (PCT, https://pct.mdanderson.org/), JAX-Clinical Knowledgebase [49], and Precision Medicine Knowledgebase (https://pmkb.weill.cornell.edu/). The tools included in the comparison and the description of their main functionalities are shown in Fig. 3a. Gene–drug associations per database are compared in Fig. 3b. PanDrugs supports single and multiple queries (i.e. gene lists) as well as standard VCF files, while DGIdb, CR, and PCT do not support variants and OncoKB, PCT, and CR do not accept multiple gene queries. To make tool comparisons viable, we selected as input only those altered genes annotated in cBioPortal.

The comparison was carried out by selecting an *EGFR*-mutant lung adenocarcinoma patient with known drug-resistance mechanisms to *EGFR* inhibitors via *MET* amplifications from the TCGA cohort (NSCLC, TCGA-38-4629). Our results show that only PanDrugs and CGI alert of the risk of a possible resistance mechanism to *EGFR* inhibitors. By contrast, the other tools offer *EGFR* inhibitors as main therapeutic options since they do not support co-occurring alterations among their functionalities.

Remarkably, only PanDrugs suggests clinically approved treatments and drugs in clinical trials for genes not considered by the other tools using biomarkers and pathway members. For instance, PanDrugs prescribes Palbociclib (Additional file 1: Figure S10A), a selective inhibitor of the cyclin-dependent kinases CDK4 and CDK6, to treat this NSCLC patient as a result of the following evidence: (1) CDK6 is a direct target; (2) CCND1, CDKN2A, and CDKN2B are biomarkers; and (iii) *CDK4* is a downstream pathway member gene. Another clear example is Navitoclax, a BCL2 family inhibitor currently tested in clinical trials for NSCLC (NCT02520778). PanDrugs suggests Navitoclax since CDK6 is a biomarker of Navitoclax response and BCL2 as pathway member because it is downstream to the *TP53* and *ERBB2* genes which are altered in this particular NSCLC patient.

Interestingly, PanDrugs is also capable of expanding drug prescription beyond known cancer genes. To illustrate this, we use the list of novel candidate cancer genes provided by Martincorena et al. PanDrugs analysis revealed 436 gene–drug associations not reported by the other tools (i.e. *MAP2K7*-Lenalidomide, *BMPR2*-Serdemetan, or *ZFP36L2*-Embelin) (Additional file 1: Figure S10B). As expected, all these associations have low GScores due to the limited clinical and biological gene annotations; however, 32 associations corresponding to 13 non-driver genes showed DScore > 0.7, suggesting their viability as potential targets for cancer treatment.

PanDrugs has been integrated within an online resource for PanCancer Analysis of Whole Genomes (PCAWG) covering 2658 donors from 48 cancer types [50]. Among these donors, we chose three patients without druggable cancer driver-altered genes from colorectal, breast, and prostate cancer to evaluate PanDrugs therapeutic proposals. The colorectal cancer patient (DO10486) showed 32 predicted damaged genes. None of the six altered drivers (*STAT3*, *SOX9*, *ARID1A*, *TGFBR2*, *RTN4*, *PPP2R1A*) are currently targeted with approved drugs although *STAT3* inhibitors are under clinical trial. PanDrugs was queried with the 32 damaged genes and proposed, among others, Dabrafenib (*LIMK1* as the direct target) and Paclitaxel (*CDK5R1* as the pathway member).

In the *TP53*-deficient breast cancer patient (DO5375) with 15 damaged genes detected (none of them known drivers), Vismodegib, an inhibitor of the Hedgehog signaling pathway, is proposed by PanDrugs as best therapeutic candidate driven by *LRP2*, a damaged gene that belongs to this pathway. Paclitaxel and Doxorubicin are also proposed by PanDrugs as the best therapeutic candidates. Interestingly, the combination of Vismodegib plus Paclitaxel and Epirubicin (an analog of Doxorubicin) is currently under clinical trial as neoadjuvant chemotherapy in triple negative breast cancer patients [https://clinicaltrials.gov/ct2/show/NCT02694224]. Eight non-driver damaged genes were found in the prostate cancer case (DO46813). Currently these genes have no drugs available to directly target them. Here, PanDrugs assigns the best DScores to approved MEK inhibitors and Vinblastine, an antitumoral alkaloid, to target pathway members downstream to the damaged *TRAF2* gene. These examples highlight PanDrugs’ capability for proposing drugs used in clinical practice in those cases with no known driver mutations and limited molecular evidence.

### PanDrugs application in a cancer case study

Unfortunately, detailed clinical annotations for patients in cancer genomics international consortiums are not publicly available to validate PanDrugs results. To overcome this limitation, we have experimentally validated PanDrugs results using a patient-derived cancer mouse xenograft (PDX) model on which several therapeutic strategies have been tested as part of a personalized medicine protocol. The protocol includes whole exome sequencing analysis and the development of PDX models as described elsewhere [51].

For this validation, our case study was a 58-year-old man diagnosed with advanced squamous cell lung carcinoma (SCLC; stage IV with brain metastasis). After surgery (R0), he received a first-line chemotherapy with carboplatin/Paclitaxel. Pemetrexed/Erlotinib was administered as a second-line therapy to treat the progression of the disease.

Tumor and normal samples of this patient were sequenced to identify tumor-specific sequence alterations. We found 965 somatic mutations and 501 somatic copy number alterations (389 gain regions and 112 loss regions) (Additional file 1: Figure S11A). We detected 318 genes that would have proteins classified as damaged by mutation consequences such as stop gains, frameshifts, and deleterious missense variants. Additional file 1: Table S8 summarizes the 46 gene mutations predicted as deleterious.

The patient’s variant list (e.g. the complete VCF file) was evaluated by PanDrugs to identify druggable genomic alterations. MAPK pathway inhibitors were suggested as the best candidates. (Additional file 1: Figure S11B). Indeed, likely underlying this suggestion, examination of the genomic events in this patient revealed deleterious somatic mutations in HRAS (G13 V), NF1 (K297*), and RAF1 (M562I) proteins. These mutations are predicted as damaging and may produce an activation of MAPK/ERK pathway. Constitutive activation of this pathway has been associated with cancers of the lung, colon, melanoma, lung, thyroid, leukemia, and pancreas [52] what makes it a suitable target to treat these tumours. MAPK inhibitors include compounds targeting MAP2K1 (MEK). Also, MAPK activity can still occur as a result of PI3K activation through RAS. Dual activation of these two pathways is observed in a number of cancer types including melanoma, prostate, and colorectal cancer, and provides the rationale for combining therapeutic agents [53].

We then performed an in vivo evaluation of the efficacy of several targeted antitumor agents—PI3K inhibitor (PI3Ki), MEK inhibitor (MEKi), rapamycin, dasatinib, and lapatinib—in a low passage PDX mouse model for this SCLC patient. Statistically significant (*p* < 0.05) tumor growth inhibition was reported for MEKi and PI3Ki treatments compared with the control group at the time point considered. Overall, benefit was reported with the combination of MEKi and PI3Ki towards the avatar model tested (Additional file 1: Figure S11C).

## Discussion

Precision oncology requires novel resources and tools to translate cancer genomic landscapes to clinical utility in order to prescribe rational, efficient, and tailored treatments to individual cancer patients [54]. The PanDrugs method has been implemented to address the interpretation gap between raw genomic data and clinical usefulness. To this end, our methodology relies on PanDrugsdb, the largest catalogue of drug-target associations currently available. This database is publicly accessible and relates druggable genes to already approved treatments, well-known targeted therapies, and preclinical drugs.

Starting from user-supplied gene or variant lists, PanDrugs identifies and prioritizes both direct or indirect targetable genomic alterations in tumors using a novel approach based on two scores: GScore and DScore. The GScore calculates target suitability for each variant (or gene) by considering its essentiality using RNAi experimental data from the Achilles project, gene relevance in cancer, tumor frequency, and the biological and clinical impacts. The DScore evaluates drug applicability by considering its clinical indications, drug status, collective gene impact, and druggability (e.g. direct target, biomarker, or pathway member) for the genes under assessment. In addition, the DScore calculation integrates in vitro drug-screening data from GDSC and CTRP. GScore and DScore are finally evaluated together to create a gene–drug ranking offering personalized candidate treatments for the variant input list. This approach estimates the treatment adequacy based on the gene–drug associations covering more biomedical sources than any other current in silico prescription tool.

Unfortunately, current anticancer therapies are based on single biomarkers that do not consider the mutational landscape of the tumor and intratumoral clonal heterogeneity [55]. Additionally, cancer genomics studies have clearly revealed that tumoral survival and progression are mainly activated by accumulation of genetic alterations in crucial molecular pathways rather than driven by single gene alterations [56, 57]. Thus, an accurate assessment of tumor fingerprints is essential for the development of effective therapies taking into account the collective gene impact and pathway context of genomic alterations from cancer patients [58, 59]. Our methodology evaluates the collective gene impact by assigning a higher DScore to that drug capable to target the highest number of genes found in the input list. PanDrugs also provides prioritized treatments beyond single direct targets and biomarkers found in variant lists by exploiting the context of pathway members. Following the druggable genome paradigm PanDrugs offers a systems biology knowledge-based layer that automatically inspects biological circuits. Interestingly, this expands cancer candidate therapies from beyond limited cancer-related gene lists to the whole druggable pathway. To our knowledge, there is no other current tool with similar characteristics.

PanDrugs-assisted therapeutic strategies have been systematically applied to large patient cohorts using TCGA patients. The feasibility of our candidate treatment proposals has been also tested in PDX experimental models. In these analyses, we found that the pathway member paradigm is able to expand in silico drug prescriptions for already approved drugs. This might have a direct impact on improving clinical decision-making by extending treatment opportunities to those patients without a clear approved pharmacogenomics biomarker.

The TCGA analysis was carried out establishing a compromise GScore threshold to retrieve highly reliable candidate treatments for well-known genes. This strategy avoids handling huge lists of results by preserving best candidates, but also discards drug–gene associations found in poorly annotated genes. However, Martincorena et al. have recently reported that half of the coding driver mutations occur outside of known cancer driver genes [7]. If this is true, precision oncology will demand the implementation of novel methodologies capable of prescribing therapies beyond known cancer genes. PanDrugs offers novel therapeutic strategies for such genes; lowering the GScore threshold while keeping the default DScore cut-off is enough to uncover reliable therapeutic options that can target poorly annotated genes. This would allow the discovery of novel clinically significant and actionable mutations that could become new genetic predictive and prognostic biomarkers.

It is important to remark that PanDrugs is more than an organized catalogue of known gene–drug relationships. PanDrugs is the first method to systematically infer novel targeted treatments following a rational framework supported by multi-gene markers, molecular pathway context, and pharmacological evidence. Our results show that in silico prescription approaches focused uniquely on known cancer genes should be complemented by incorporating drug information associated to genomic alterations located in non-cancer genes. Our approach extends the treatment opportunities of cancer patients by enriching the therapeutic arsenal against tumors and opens new avenues for personalized medicine.

Although PanDrugs offers a valuable methodology for in silico prescription, further efforts are required to improve cancer treatment by proposing more effective drugs and anticipating drug resistance. Since drug efficacy substantially depends on tissue-, cell-, and molecular-specific context [60], precision oncology tools should integrate data beyond pure genomics [61] including the combination of high-throughput drug screenings and functional experiments to unravel heterogeneous multi-omic dependencies influencing response to therapy [62, 63]. In addition, the integration of additional biological relationship layers such as protein interaction networks [64], transcriptional regulatory modules [65], or pathway activity footprints [66] should improve drug prioritization and will help to propose alternative therapeutic strategies. It is also crucial to have a comprehensive and well-structured drug ontology available that provides drug annotations (i.e. drug indication, mechanisms of action, chemical structure, side effects, drug-target associations, and drug families) for a more accurate drug prescription [67].

Finally, it should be emphasized that current in silico drug prescription tools are limited by the lack of large longitudinal precision medicine studies with accessible clinical records. Such studies are crucial to assess and validate drug proposals and refine in silico prescription algorithms to consider additional factors such as mode of drug administration, combinatorial therapies, drug repositioning, and side effects.

## Conclusions

PanDrugs provides a feasible method to guide genomic-hypothesis therapies as well as to prioritize multiple druggable alterations in genomically complex tumors. Indeed, PanDrugs represents the first drug prescription tool that proposes cancer therapies with a rationale based on pathway context, collective gene impact, and information provided by functional experiments. PanDrugs has demonstrated its adaptability by being systematically applied to large cohorts of patients and by providing candidate treatments directed to druggable genes beyond cancer driver genes. Overall, our method highlights new areas of opportunity for advancing precision cancer medicine, providing a novel and fully accessible method that could be useful in decreasing the complexity of the interpretation of genomic data and clinical decision-making. PanDrugs is freely available at http://www.pandrugs.org.

## Availability and requirements

Project name: PanDrugs.

Project home page: http://www.pandrugs.org

Operating system(s): Platform independent.

Programming language: Java, MySQL RDBMS, Perl.

Other requirements: Chrome, Firefox, Safari.

License: GPLv3.

## Additional file

Additional file 1: Supplementary materials and methods, Figures S1–S11 and Tables S1–S8. (PDF 14845 kb)

## Abbreviations

CGI: Cancer Genome Interpreter
CR: CancerResource
NSCLC: Non-small cell lung cancer
PCT: Personalized cancer therapy
PDX: Patient-derived xenograft
PMKB: Precision Medicine Knowledgebase
RNAi: RNA interference
SCLC: Small-cell lung cancer
TCGA: The Cancer Genome Atlas
TKI: Tyrosine kinase inhibitor
VCF: Variant call format
