# Supplementary material for: PanDrugs: a novel method to prioritize anticancer drug treatments according to individual genomic data
Source: Genome Med. 2018 May 31;10:41. doi: 10.1186/s13073-018-0546-1 (PMC5977747; doi:10.1186/s13073-018-0546-1)
Supplement: Supplementary file 1 — Supplementary materials and methods, Figures S1–S11 and Tables S1–S8. (PDF 14845 kb) [file 13073_2018_546_MOESM1_ESM.pdf]

SUPPLEMENTARY MATERIALS AND METHODS

PanDrugs: a novel method to prioritize anticancer drug treatments according to individual genomic data

Elena Piñeiro-Yáñez, Miguel Reboiro-Jato, Gonzalo Gómez-López, Javier Perales-Patón, Kevin Troulé, José Manuel Rodríguez, Héctor Tejero, Takeshi Shimamura, Pedro Pablo López-Casas, Julián Carretero, Alfonso Valencia, Manuel Hidalgo, Daniel Glez-Peña, Fátima Al-Shahrour

Table of Contents

SUPPLEMENTARY TEXT……..2
MATERIAL AND METHODS...….....……………………………………………………………..2
Drug-Gene data sources..……………………………………………………………………………...2
Drug name standardization..…………………………………………………………………….....3
Gene and drug annotations ………………………………………………………………………....3
Data source integration ……………………………………..……………………………………….5
Gene Score (GScore) calculation..………………………………………………………………….6
Drug Score (DScore) calculation ……………………………………………………………………..7
PanDrugs software implementation …………………………………………………………………….8
RESULTS...………………...………………………………..……………………………………....8
Analysis in TCGA data ..…………………………………..…………………………………………...8
Application in a cancer case study..……………………………………….……………………10
SUPPLEMENTARY REFERENCES…...………………………………………………………11
SUPPLEMENTARY FIGURES...13
Supplementary Figure S1 ………...13
Supplementary Figure S2 ………...14
Supplementary Figure S3 ………...15
Supplementary Figure S4 ………...16
Supplementary Figure S5 ………...17
Supplementary Figure S6…...........……...18
Supplementary Figure S7 ………...19
Supplementary Figure S8…..........……..20
Supplementary Figure S9…......................……...21
Supplementary Figure S10..............................……...22
Supplementary Figure S11..............................……...23
SUPPLEMENTARY TABLES ……….. 24
Supplementary Table 1... 24
Supplementary Table 2... 25
Supplementary Table 3... 26
Supplementary Table 4... 27
Supplementary Table 5... 28
Supplementary Table 6... 29
Supplementary Table 7... 30
Supplementary Table 8... 34

SUPPLEMENTARY TEXT

MATERIAL AND METHODS

Drug-Gene data sources

In order to construct Pandrugs database (PanDrugsdb), we have collected and integrated relationships between drugs and genes from 18 sources with different origins and levels of information including data from experimental studies in cancer cell lines (Supplementary Table 1):

DGIdb: DGIdb [1] constitutes a comprehensive catalogue of information about gene druggability. They classify this information into two main categories: i) known drug-gene associations from databases and literature, and ii) potentially druggable genes based on their belonging to a particular druggable gene category. To build PanDrugsdb, we focused in the first kind of information that DGIdb mine from several resources (Cancer Commons, Cancer Genome Interpreter (CGI), ChemblInteractions, CIViC, CKB, Clearity Foundation, DoCM, DrugBank, FDA, Guide to PHARMACOLOGY, My Cancer Genome, NCI, OncoKB, PharmGKB, TTD and the information in the TALC, TDG and TEND studies). We accessed DGIdb using its Application Programming Interface (API) to retrieve the drugs associated with all the human genes.

Monoclonal antibodies: Targeted therapy with monoclonal antibodies is highly selective and has being established as a successful treatment in several diseases and specially in cancer. For this reason we decided to incorporate this information in PanDrugsdb. Most of this information derives from the list of therapeutic monoclonal antibodies (moAb) from Carter and Lazar [2].

TARGET: Another source of information is the tumor alterations relevant for genomics-driven therapy (TARGET) database [3] supported by the Cancer Genome Analysis of the Broad Institute, which includes genes somatically altered in cancer, associated with clinical actions in a standard spreadsheet file. We downloaded the last version available at the moment of the query (TARGET_db_v3_02142015.xlsx). We selected among the records those with a specific drug name, extracting also the additional information they provide about the sensitivity or resistance response, the type of drug-gene relation understood as a drug target or biomarker and the type of genomic alteration associated to the drug response.

Cancer Therapeutics Response Portal (CTRP): In this study [4,5], corresponding to the CTRP V1, the authors measure the sensitivity of 242 genomically characterized cancer cell lines to a set of 354 small molecules in different approval status (approved, clinical candidates and probes). The information about interactions was extracted from the supplementary file 2 of the article (mmc2.xlsx). We selected the significant drug-gene interactions using as threshold q-value = 0.05. Both sensitivity and unresponsive were maintained. We filtered out from the list that ambiguous information where both types of response (sensitivity or unresponsive) appear related to the alteration of a particular gene.

Genomics of Drug Sensitivity in Cancer (GDSC): In this study [6], the authors map the genomic alterations detected in a large-scale cancer study with the annotations from different cancer cell lines, where they have measured the sensitivity response to several anticancer drugs and linked this response to the genomic information. We downloaded the file TableS4C.xlsx from Iorio and collaborators [6] which contains the significant results of the multivariate ANOVA test used in the experiments. The information in this file is stratified by the different tumor types but also for the global PanCancer set. We extracted the ANOVA results for the PanCancer data set.

Drug name standardization

We used the PubChem Identifier Exchange Service from PubChem resource [7] (accessed on 15th February 2018) where a list of synonyms is provided for a particular compound. For each of the returned alternative names we selected the first one, that is then used as the standard name. Next step consisted on a manual revision to correct possible inconsistencies in the standardization and to assign a standard name to those cases in which there were no entry in PubChem. To obtain the show name we retrieved the file ligands.csv from the Guide to PHARMACOLOGY [8] version 2017.6 where the International Nonproprietary Name (INN) was extracted. This process was followed by manual curation to revert possible inconsistencies.

Since different databases can use alternative names to mention the same compound, drug names were standardized in order to be consistently integrated in PanDrugsdb. There is no clear consensus for the huge range of available synonyms and, in some cases, they are ambiguous and employed in a wrong way. To avoid the complexity in some of the standardized names we provide an alternative name when showing the final results. That is the INN or the source name when this is not available or is the standardized name is too long (which usually happens with chemical nomenclatures of compounds).

Gene and drug annotations

KEGG pathways: We obtained a list of pathway's codes linked to the involved genes from Kyoto Encyclopaedia of Genes and Genomes (KEGG) pathways database [9]. For this purpose we used the REST API available in the release 85.1 of this database. This list was used to map the genes in PanDrugsdb with the pathways they are involved in.

Drug Family: To define the family for the drugs we used two sources. One is the Target-based Classification of Drugs from KEGG (release 85.1). This resource provides a hierarchical classification for each drug with several terms that go from the most general to the most specific. To assign a unique category, we chose the second most general term. The other resource is the classification of drugs established in the Connectivity Map (CMap) [10]. We obtained a file with a list of chemicals considered in the CMap resource and the linked Mechanism of Action from the Repurposing section of Clue.io portal for CMap project (accessed on February 2018).

Drug status: We used the drug information from FDA [11] and information about clinical trials [12] (February of 2018). The drug status was manually curated in order to identify the current cancer therapies and the therapies that could be used in a repositioning way. Following categories and subcategories were distinguished (Supplementary Figure S2A):

1. Approved:
   a. In cancer: When the drug appears approved by the FDA and it is indicated in a cancer treatment. The cancer type and the therapy type were also incorporated into the database.
   b. In cancer clinical trials: When the drug appears approved by the FDA and it is used in other conditions different from cancer, but it is under study in a cancer clinical trial as a potential treatment.
   c. Other: When the drug appears approved by the FDA and it is used in other conditions different from cancer.
2. Clinical trials:
   a. In cancer clinical trials: When the drug does not appear approved by the FDA but it is under study in a cancer clinical trial as a potential treatment.
   b. Other: When the drug does not appear approved by the FDA but it is under study in a cancer clinical trial for a different pathology from cancer.
3. Experimental: When the drug is in a pre-clinical stage.
4. Withdrawn: When the compound appears as withdrawn in the FDA.
5. Undefined: When the drug name refers to a set of compounds but not to a specific one.

Pathological area: This information was obtained from FDA labels, classifying the different indications into at least one of the defined categories in Supplementary Figure S2B. For approved drugs in cancer, compounds are encompassed in groups depending on the anatomic location of the tumor type they are prescribed for (Supplementary Figure S2C).

Definition of direct target or biomarker gene in the drug response: In each drug-gene association, the gene can have a different role in the drug response. We call direct target to a gene that contributes to a disease phenotype and can be directly targeted by a drug (small molecule, monoclonal antibody...). For example, EGFR in the use of an EGFR tyrosine kinase inhibitor (TKi). In the other hand, we call biomarker to a gene which genetic status is associated with a drug response by clinical or pre-clinical evidences but its protein product is not the direct target of the drug. For example, MET in the use of EGFR TKi, where amplifications in this gene cause a resistance response to these compounds. To assign the target/biomarker label to each relation we have taken into account the type of information stored in the different databases (Supplementary Table 1). Cancer Commons, CGI, Clearity Foundation Clinical Biomarkers, DrugBank, FDA, Guide To PHARMACOLOGY, My Cancer Genome, NCI, TALC, Tdg Clinical Trial, TEND, TTD from DGIdb or additional sources of monoclonal antibodies, discarding some controlled exception, store associations where the gene is the target of the drug. We added to the target set, those records in TARGET database where the gene is stated as target and also well-known associations described as target gene-drug. The remaining drug-gene associations were labelled as biomarker. In its current version PanDrugsdb supports biomarkers such as gene mutations, amplifications, deletions, gene fusions, alterations in gene expression and promoter methylation modifications reported to drive resistance or sensitivity in response to drugs.

Resistance/Sensitivity drug response: This information was obtained from CGI, CIViC, CKB, My Cancer Genome, My Cancer Genome Clinical Trial, Clearity Foundation Biomarkers, DoCM, FDA, NCI, TALC, TARGET, GDSC and CTRP that store drug response information. Relations for which information was not available were indicated as sensitivity (Supplementary Table 1).

Molecular alteration type: The definition of the molecular alteration type of the drug-gene associations was performed by a combination of the existing information in CGI, CIViC, CKB, Clearity Foundation Biomarkers, DoCM, FDA, NCI, OncoKB and TARGET (Supplementary Table 1) along with a manual curation based on the existing knowledge in the literature. The types of driven molecular alterations included were missense mutation, amplification, deletion, gene fusion, gene expression dysregulation and promoter methylation.

Data source integration

Drug-gene association data was manually downloaded from the different resources. This data was automatically parsed using a combination of custom python and perl scripts when possible, or mixed with a manual intervention in those cases that require a more exhaustive edition. This step created a tabular plain text for each of them. Then, using a perl module, these files were combined, and for each drug-gene interaction, all the corresponding annotations were incorporated and a pre-computed drug score was calculated for each of them.

All records in this database were joined (Supplementary Figure S2D) keeping only one record for the quartet gene-source-original name-standard name, because of the duplications that can appear in some files. In most of the sources, there are cases in which there are several records with different original names that converge in a unique standard name. These records were kept and they were not unified to control the presence of inconsistencies in the drug name standardization process. In any case, they are shown as a unique assignation when suggesting drugs. We finally obtained the PanDrugsdb with 9092 unique drugs, 4804 unique genes and 43909 unique drug-gene interactions. Distribution of this final drug-gene associations is represented in (Supplementary Figure S2E).

Gene Score (GScore) calculation

The Gene Score value (GScore) ranges between 0 and 1 and it allows the prioritization at gene level taking into account the biological relevance of the gene in carcinogenesis and the therapeutic actionability. GScore calculation depends on the provided input type (gene list or VCF file) and is weighted depending on the level of gene association with cancer. If PanDrugs input is a VCF file the GScore will be computed taking into account the information provided by the variants located in each gene resulting in a gene prioritization based on variant information.

1. *List of gene symbols*: The GScore is calculated as shown in the global formula expressed in Supplementary Figure S3A and Supplementary Table 2. To perform

this calculation we consider four evidences: i) the frequency at which the gene appears in different tumors, ii) the probability of being a cancer driver, iii) the gene essentiality based on RNA silencing studies in cancer cell lines, iv) and the oncogenic score of genes based on the integrative analysis of OncoScape [13]. Each of these evidences has an associated weight.

a. To evaluate the frequency of appearance in carcinogenic processes we used two information sources. On one hand, we use the list of genes in the Cancer Gene Census (CGC) of COSMIC v84 [14] and also the TumorPortal resource [15], assigning a different weight according to the frequency at which the gene is altered in any tumor type. Within TumorPortal, they establish three categories: *Highly significantly mutated, Significantly mutated* and *Near significance* ordered from high to low mutation frequency. A decreasing weight is provided for each of them.
b. To score the probability of being a tumor driver, we use the information obtained from Tamborero et al. [16] that identify a set of potential tumor drivers using mutational information from TCGA. The weight given by this component will depend on the assigned probability in the study (*High Confidence Driver* or *Candidate Driver*).
c. To calculate the essentiality score genes were ranked by the negative Pearson's correlation between the phenotype value calculated by ATARiS algorithm [17] and the gene expression value from all the cancer cell lines in common from the two datasets (n=216) (the Project Achilles [18] and the CCLE data [19]). Thus, the higher is the gene expressions and the lower is the phenotype values in cancer cell lines for a given gene, the more essential is the gene. A similar approach was used in the original study by Shao et al. with 83 cancer cell lines. The resulting ranking of correlations were transformed using a min-max normalization into a continuous 0-1 range.
d. OncoScape is a method to identify cancer candidate genes by the integration of different molecular data from 11 cancer types. It integrates information about gene expression data, somatic mutations, DNA copy-number variation, methylation and data from shRNA knock-down screens, this last particularly interesting for our approach. The scores provided in OncoScape were used as a base for our GScore weighting.

2. *VCF file*: We first execute the variant effect predictor of ensembl (VEP) [20]. This tool provides annotations and predictions for the variants that we after enrich with additional information. We keep the variants with an important impact in the transcriptional process (VEP consequence equal to transcript_ablation, splice_donor_variant, splice_acceptor_variant, stop_gained, frameshift_variant, stop_lost, start_lost, transcript_amplification, inframe_insertion, inframe_deletion, missense_variant, protein_altering_variant, splice_region_variant, incomplete_terminal_codon_variant and stop_retained_variant). The score for each variant is called VScore and is computed as shown in the Supplementary Table 3. Each contribution is conditioned by the role assigned to the gene. To decide the role we use the consensual information of CGC and the prediction made by oncodriveROLE [21]. The genes not present in any of them or that have opposite role labels in each resource are evaluated to see the consequence of

the variant. If the consequence is a stop gain, stop lost, a frameshift or a splice alteration, they are labeled as tumor suppressors. The remaining variants for which we cannot assign a label are graded in the same way as oncogenes. Among all the calculated VScores of a particular gene, we select the highest and establish it as the GScore. Only the most relevant transcripts according to the criteria established by APPRIS [22] are taken into account in the selection.

3. *Ranked list of genes:* The input can be a ranked list of genes based on some experimental results, as for example, data originating from differential expression studies. The provided values are normalized to the 0-1 scale using min-max scaling.

Drug Score (DScore) calculation

The Drug Score (DScore) allows to prioritize the suggested therapies and reflects the suitability of a treatment according to the genomic profile. It goes from -1 to 1 with the negative values corresponding to resistance and positive values corresponding to sensitivity (Supplementary Figure S3B). We have a precomputed DScore in the database which is based on each single drug-gene relation. In its calculation we first take into account the use of the drug in cancer, then the approval status of the drug and finally the definition of the gene as a target or marker in the relation with the drug. Experimental compounds have a different score assignation, but they rank below drugs in another status, giving more relevance to target than to biomarker genes.

PanDrugs assignation process

PanDrugs gene-drug assignation process consists on the generation of a ranked list of drugs associated to the input gene list. In this assignation process PanDrugs suggests drugs in two ways: 1) directly and 2) following pathway-member paradigm. In the direct way PanDrugs search for drugs against direct targets or biomarkers. The pathway member approach allows to expand the therapeutic options and consists on gene-drug assignations where the drug target is a gene located downstream to the altered one.

For pathway member search we built a catalogue of biological pathways involved in a variety of processes related to cancer. To do so, we extracted from the modelled pathways available in hiPathia [23] all the possible subpathways comprising four nodes at maximum. Only those nodes categorized as ‘gene’ were considered, while other nodes (i.e. glycans) were discarded. Treatment options suggested for these nodes(genes) depends on: i) the functional role of the nodes in cancer (oncogene, tumor suppressor gene or dual role of oncogene and tumor suppressor gene) and ii) the type of interaction (activation or inhibition) with the child node.

During the assignation process the precomputed DScore is adjusted to take into account the information provided by the input data collectively. We define the ‘collective gene impact’ by assigning a higher DScore to that drug capable to target the highest number of genes found in the input list. We also consider the number of expert curated databases supporting a particular drug-gene relationship. Indirect drug-gene relationships are penalized with respect

to direct ones unless a 'biomarker' evidence supports the association. Experimental drugs have no DScore readjustment maintaining the precomputed DScore and only penalizing the indirect cases (Supplementary Figure S3C). If one particular drug has a sensitivity response due to one gene, but a resistance response due to another, the drug response assignation will be "Both". The drug reference assignation employs the sign of the highest DScore in absolute value (max |DScore|). This means that the drug response with the highest evidence is the one that is going to be selected to allocate the drug in the sensitivity or resistance area by default. Full details regarding sensitivity and resistance for each particular case are downloadable and accessible in PanDrugs through pull-down menus.

PanDrugs software implementation

The back-end application is in charge of (i) storing gene and drugs data, (ii) perform and manage genomic variants analyses and (iii) allow external applications to access the data and services through a public REST API. The database is stored in the MySQL RDBMS. Variant analyses are performed with a Perl script which computes the scores for the mutated genes from user-provided VCF file. A previous annotation step with the VEP release 90 is performed. In order to manage multiple variant analysis simultaneously a Java scheduling program using a thread pool was implemented. The REST API allows external programs to query the PanDrugsdb over HTTP. It was implemented in Java with the JAX-RS API. Both the scheduling program and the REST API service runs in a single Java EE application in Apache Tomcat 8.

The front-end application in is charge of get user queries, communicate with the backend REST API and display results in an user-friendly interface. This application is implemented with AngularJS 1.4. The Highcharts library is used for the Gene and Drugs score chart and the D3 library for the visualization of PanDrugs across TCGA tumoral landscape.

RESULTS AND DISCUSSION

Analysis in TCGA data

Mutations and CNVs data employed this study was obtained from 20 different tumor types available in TCGA project. The workflow followed for this analysis is represented in Supplementary Figure S5.

For mutation data, we took the MAF files from synapse syn1729383 (Supplementary Table 4). In particular, we selected the cleaned_filtered.maf file with the whole filtered alterations for 19 tumor types (COAD and READ are concatenated) and to speed up the process we removed those genes that do not appear in PanDrugsdb. Then, we calculated the GScore for each gene based on variant information. To do this, we ran the VEP release 90 using the corresponding cache files of the human genome (hg19 version) over an ensembl-format converted file version of the mutations of the MAF files. From the resulting files, we kept those variants with high impact consequence (transcript_ablation, splice_donor_variant, splice_acceptor_variant, stop_gained, frameshift_variant, stop_lost, start_lost, transcript_amplification, inframe_insertion, inframe_deletion, missense_variant,

protein_altering_variant, splice_region_variant, incomplete_terminal_codon_variant and stop_retained_variant) and we added the annotations used to compute our VScore. Then, from these VScores, the GScore for each gene was established as described for VCF input files. Databases and the corresponding versions used in this process are indicated in Supplementary Table 5.

For CNVs, we took the file gistic.all_thresholded.by_genes from synapse (Supplementary Table 4) for each of the tumor types. The GScore was calculated in this case according to a gene level criteria as described above for a list of genes. We kept those records with CNVs defined as -2 (homozygous deletion), 2 (high level amplification), -1 (heterozygous deletion).

In order to establish a suitable threshold to filter genomic events with an unclear functional effect, we performed several filtering steps using increasing values of GScores for both mutations as CNVs. As can be observed in the Supplementary Figure S6A, above a GScore of 0.4 the number of genes with a mutation event drops. Thus, establishing a gene score threshold above 0.4 would remove the bulk of alterations that in principle would not contribute to the pathogenic process. For CNVs the decrease was observed at low threshold values of GScore and it presents a more continuous pattern in the reduction of the number of genes.

Looking at the same distribution and considering the number of patients instead of the number of genes (Supplementary Figure 6B), the selection of this threshold would still cover a great number of patients. The selected CNV events would be present in a large number of patients in comparison with SNVs despite of being significantly reduced.

Taking into account the complementarity of both events (Supplementary Table 6 and Supplementary Figure S6C), from an initial number of 7096 cases, more than 6000 would still maintain some mutational and/or CNV events that could be used to identify potential therapies. Distribution of these events affecting genes EGFR and KRAS across the 20 tumor types of these study can be seen in the (Supplementary Figure S7)

Mutations and CNV data was integrated and affected genes were queried against PanDrugs for each TCGA patient. In this query oncogenes and tumor suppressor genes were managed in a different way. Treatments for oncogenes were provided directly (direct targets) and through pathway members (for KRAS mutant patients MEK inhibitors proposed as pathway members were excluded). However, for tumor suppressor genes, only indirect treatments through pathways members were considered. To determine the behaviour of the gene as oncogene or tumor suppressor gene in this context, where mutational and CNV data are integrated, we considered additional guidelines. If there was a mutation with a role information different from unclassified (when no assumption could be made about the role) in an affected gene, this role was assumed as the role of the gene. Otherwise, and when there were CNVs affecting the gene, it was considered as oncogene if it was affected by an amplification or tumor suppressor gene if it was affected by a deletion. If after this process the role of the gene remained unclassified, the label established in most of the cases for that gene in the patients for that particular tumor type was assigned. Those genes for which it was not possible to determine a role of oncogene or tumor suppressor were queried in both direct and pathway member. Most frequently altered genes suggested for treatment by PanDrugs in TCGA patients are indicated in Supplementary Table 7.

We compared PanDrugs performance applying our methodology to the TCGA cohort previously used in Rubio-Perez C. et al. study [24]. To analyze this data, we focused on the common patients in our TCGA analysis and the core cohort exclusively integrated by the TCGA cases of the Rubio-Perez C. et al. analysis. To that end, we used the information provided in the supplementary material S4D from Rubio-Perez et al manuscript to make the comparison and extracted those cases present in our TCGA study. To make both sets comparable we reduced the alterations detected with our processing to those considered drivers in each tumor type. Drug assignments were then recalculated and represented without filtering by GScore. Only those drugs without evidences of resistance and supported by at least two sources are contemplated.

Application in a cancer case study

WES analysis: Tumoral and normal samples of this patient were sequenced to identify tumor-specific sequence alterations. Exome variant analysis and biological impact predictions were performed by RUbioSeq software [25] using default parameters for somatic variation analysis. In detail, sequencing data were first analysed by FastQC for quality control inspection and then aligned to the human reference genome (GRCh37) using Burrows-Wheeler alignment (BWA-MEM) [26]. Somatic variants were identified using the HaplotypeCaller available at the GATK [27]. For variant calling we used GATK HaplotypeCaller with default parameters for filtering. Biological impact predictions for detected variants were obtained from VEP. In order to estimate the biological impact of the missense mutations, we used different algorithms (i.e. SIFT, PolyPhen-2, CONDEL, Pfam, InterPro, etc.). Then, other GScore annotation sources (cancer essentiality, relevance in cancer, frequency and clinical implication) are incorporated and GScore itself is calculated to rank selected somatic variants.

Drug efficacy testing in PDX: We evaluated the antitumor activity of a MEK inhibitor (MEKi), a PI3K inhibitor (PI3Ki), an mTOR inhibitor (rapamycin), a multi- BCR/ABL and Src family tyrosine kinase inhibitor (dasatinib) and a HER2 inhibitor (lapatinib), in a low passage lung cancer (squamous cell carcinoma) patient-derived xenograft (PDX) mouse model. Data collected from this study includes animal weights, tumor dimensions and daily observations; this information was used to determine anticancer activity based on tumor growth inhibition or regression. The designated endpoint for this study was a mean control tumor volume of approximately 1.5 cm^3. Animals were implanted with tumor fragments harvested from host animals and the study initiated up to 50 days later at an average tumor volume of approximately 200 mm^3. No tumor burden was associated with this model based on lack of weight loss or animal morbidity in the control group.

In this study, the doses used with all drugs were well tolerated with no significant (≥ 20%) weight loss reported in any group. Statistically significant ($p<0.05$) tumor growth inhibition was reported for MEKi and PI3Ki treatments compared with control group at the time point considered.

SUPPLEMENTARY REFERENCES

1. Wagner AH *et al*. DGIdb 2.0: mining clinically relevant drug-gene interactions. Nucleic Acids Res 44 (D1), D1036-44 (2016).
2. Carter PJ & Lazar GA. Next generation antibody drugs: pursuit of the 'high-hanging fruit'. Nat Rev Drug Discov. 2017 Dec 1.
3. Van Allen, EM. *et al*. Whole-exome sequencing and clinical interpretation of formalin-fixed, paraffin-embedded tumor samples to guide precision cancer medicine. Nat Med 20 (6), 682-688 (2014).
4. Basu, A. *et al.* An interactive resource to identify cancer genetic and lineage dependencies targeted by small molecules. Cell 154 (5), 1151-1161 (2013).
5. Rees, MG. *et al.* Correlating chemical sensitivity and basal gene expression reveals mechanism of action. Nat Chem Biol 12 (2), 109-116 (2016).
6. Iorio, F. *et al.*, A Landscape of Pharmacogenomic Interactions in Cancer. Cell 166 (3), 740-754 (2016).
7. Kim, S. *et al.*, PubChem Substance and Compound databases. Nucleic Acids Res 44 (D1), D1202-1213 (2016).
8. http://www.guidetopharmacology.org
9. http://www.genome.jp/kegg/
10. Lamb, J. The Connectivity Map: a new tool for biomedical research. Nat Rev Cancer 7 (1), 54-60 (2007).
11. http://www.accessdata.fda.gov/scripts/cder/drugsatfda/
12. https://clinicaltrials.gov/
13. Schlicker, A. *et al.*, OncoScape: Exploring the cancer aberration landscape by genomic data fusion. Sci Rep 6, 28103 (2016).
14. Futreal, PA *et al.*, A census of human cancer genes. Nat Rev Cancer 4 (3), 177-183 (2004).
15. Lawrence, MS. *et al.*, Discovery and saturation analysis of cancer genes across 21 tumour types. Nature 505 (7484), 495-501 (2014).
16. Tamborero, D. *et al.*, Comprehensive identification of mutational cancer driver genes across 12 tumor types. Sci Rep 3. 2650 (2013).
17. Shao, DD. *et al.*, ATARiS: computational quantification of gene suppression phenotypes from multisample RNAi screens. Genome Res 23 (4), 665-678 (2013).
18. Cowley, GS. *et al.*, Parallel genome-scale loss of function screens in 216 cancer cell lines for the identification of context-specific genetic dependencies. Sci Data 1, 140035 (2014).
19. Barretina, J. *et al.*, The Cancer Cell Line Encyclopedia enables predictive modelling of anticancer drug sensitivity. Nature 483 (7391), 603-607 (2012).
20. McLaren, W. *et al.*, Deriving the consequences of genomic variants with the Ensembl API and SNP Effect Predictor. Bioinformatics 26 (16), 2069-2070 (2010).
21. Schroeder, MP. *et al.*, OncodriveROLE classifies cancer driver genes in loss of function and activating mode of action. Bioinformatics 30 (17), i549-555 (2014).
22. Rodriguez, JM. *et al.*, APPRIS WebServer and WebServices. Nucleic Acids Res 43 (W1), W455-459 (2015).
23. https://gitlab.com/groups/hipathia-code

24. Rubio-Perez C, Tamborero D, Schroeder MP, Antolín AA, Deu-Pons J, Perez-Llamas C et al. In silico prescription of anticancer drugs to cohorts of 28 tumor types reveals targeting opportunities. Cancer Cell. 2015; 27:3; 382-96.
25. Rubio-Camarillo, M. *et al.*, RUbioSeq: a suite of parallelized pipelines to automate exome variation and bisulfite-seq analyses. Bioinformatics 29 (13), 1687-1689 (2013).
26. Li, H. and R. Durbin, Fast and accurate short read alignment with Burrows-Wheeler transform. Bioinformatics, 2009. 25(14): p. 1754-60.
27. DePristo, M.A., et al., A framework for variation discovery and genotyping using next-generation DNA sequencing data. Nat Genet. 43(5): p. 491-8.

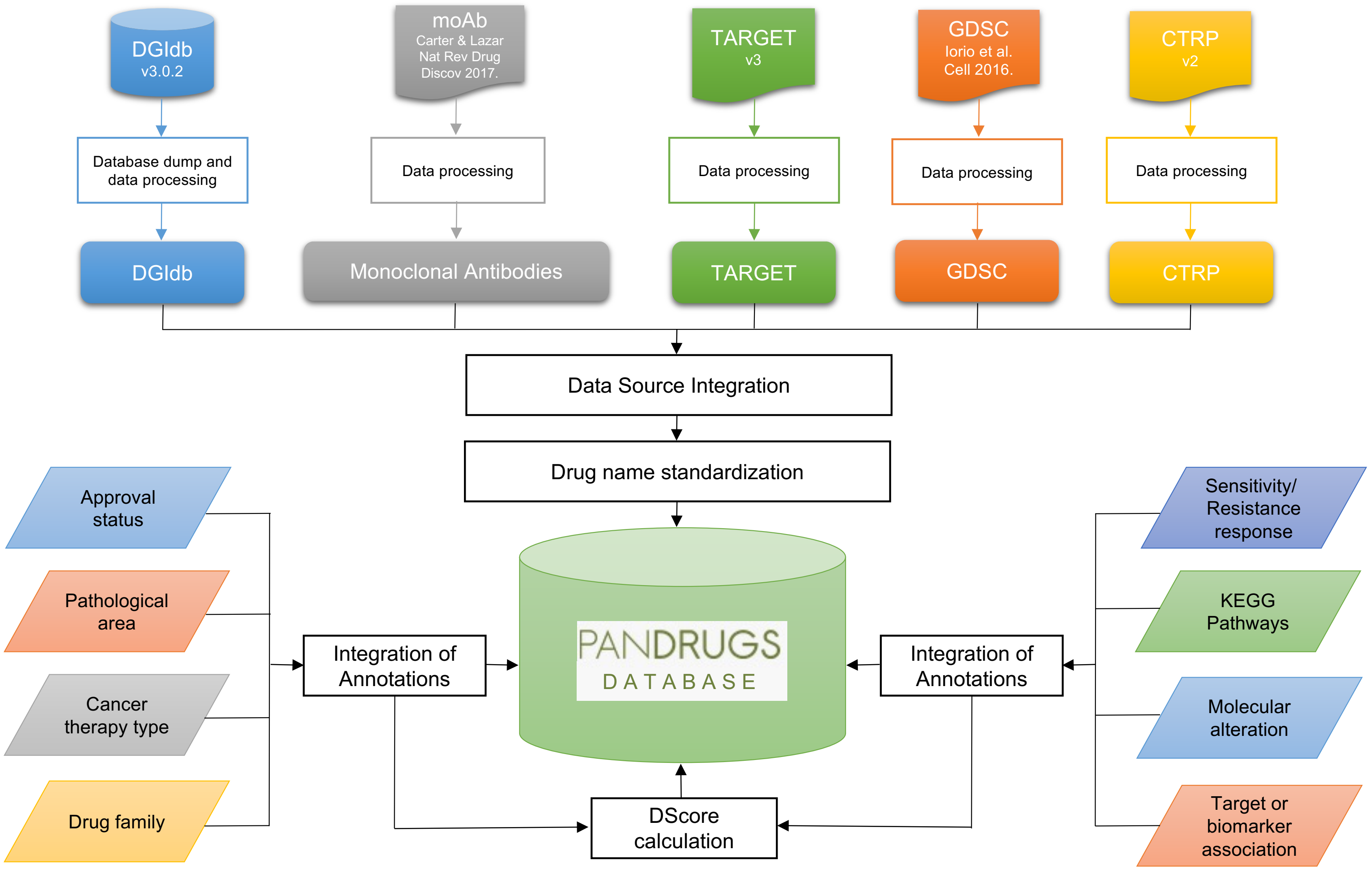

Supplementary Figure S1. PanDrugs database integration schema.

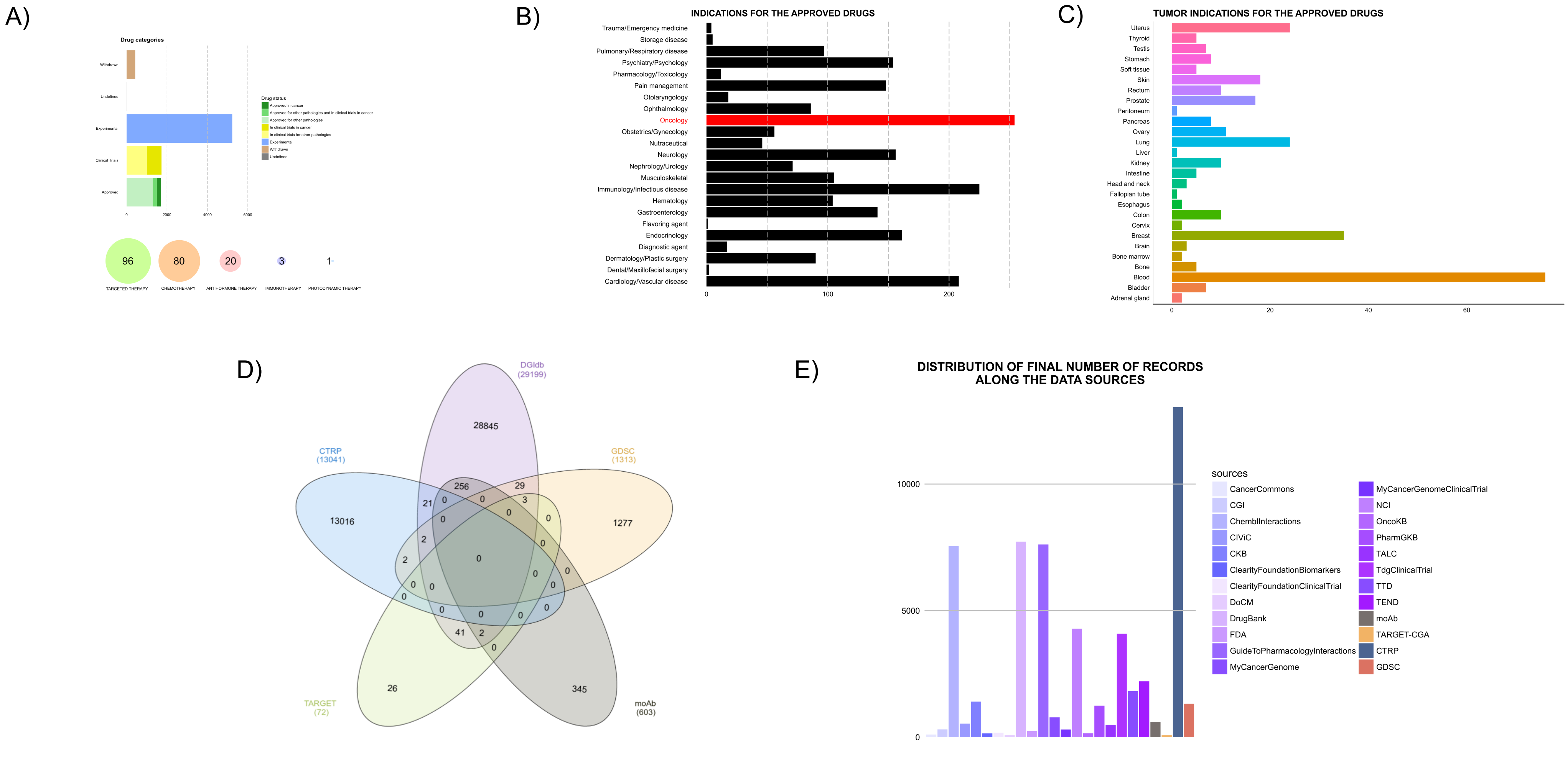

Supplementary Figure S2. A) Bar-plot showing commercial status of drugs available in PanDrugs. Circle size represents the number of anticancer drugs in PanDrugs sorted by therapy type. B) Bar-plot of medical indications for the approved drugs available in PanDrugs. C) Bar-plots of tumor indications for the approved drugs available in PanDrugs. D) Overlap amongst gene-drugs records in data sources employed in PanDrugs. E) Bar-chart showing the gene-drug record distributions according to their source of origin (redundancies in the different sources of DGIdb have been removed).

A)

GScore = [0.4 * Essentiality Score] + [0.3 * (max {max OncoScape score for oncogene in different tumor types, max OncoScape score for tumor suppressor gene in different tumor types} normalized between 0 and 1)]

+ [Presence in cancer (Tumor Portal)
0.1 (If *Highly significantly mutated*)
0.05 (If *Significantly mutated*)
0.025 (If *Near Significance*)] + [Presence in cancer (CGC)
0.1 (If *present in CGC*)] + [cancer driver
0.1 (If *High Confidence Driver*)
0.05 (If *Candidate Driver*)]

B)

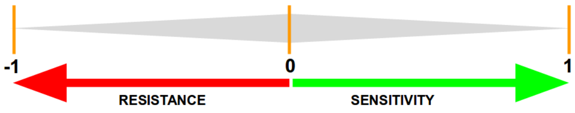

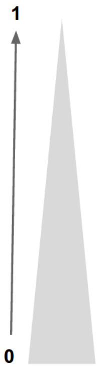

DISEASE	DRUG STATUS	DRUGGABLE GENE	SCORE
Cancer	Approved	Direct target	± 1
		Biomarker	± 0.9
Other disease (Clinical trials in cancer)		Direct target	± 0.8
		Biomarker	± 0.7
Cancer	Clinical trials	Direct target	± 0.6
		Biomarker	± 0.5
Others	Approved	Direct target	± 0.4
		Biomarker	± 0.3
	Clinical trials	Direct target	± 0.2
		Biomarker	± 0.1
	Experimental	Direct target	± 0.0008
		Biomarker	± 0.0004

C)

Approved and Clinical trials drugs:

Pre-computed DScore value = [Cancer + Drug Status + Druggable gene type]*[if *resistance* (-1)]
Collective gene impact = # genes (max. 9) + [-1(if pathway member)]
Database factor = # expert curated sources (max. 9)

DScore = max{Pre-computed DScore value} - 0.1 + (0.01 * Collective gene impact) + 0.001 + (0.001 * Database factor)

Experimental:

DScore = max{Pre-computed DScore value} - 0.0002 (if indirect)

Supplementary Figure S3. A) GScore calculation. B) Features contributing to pre-computed DScore calculation. C) DScore calculation.

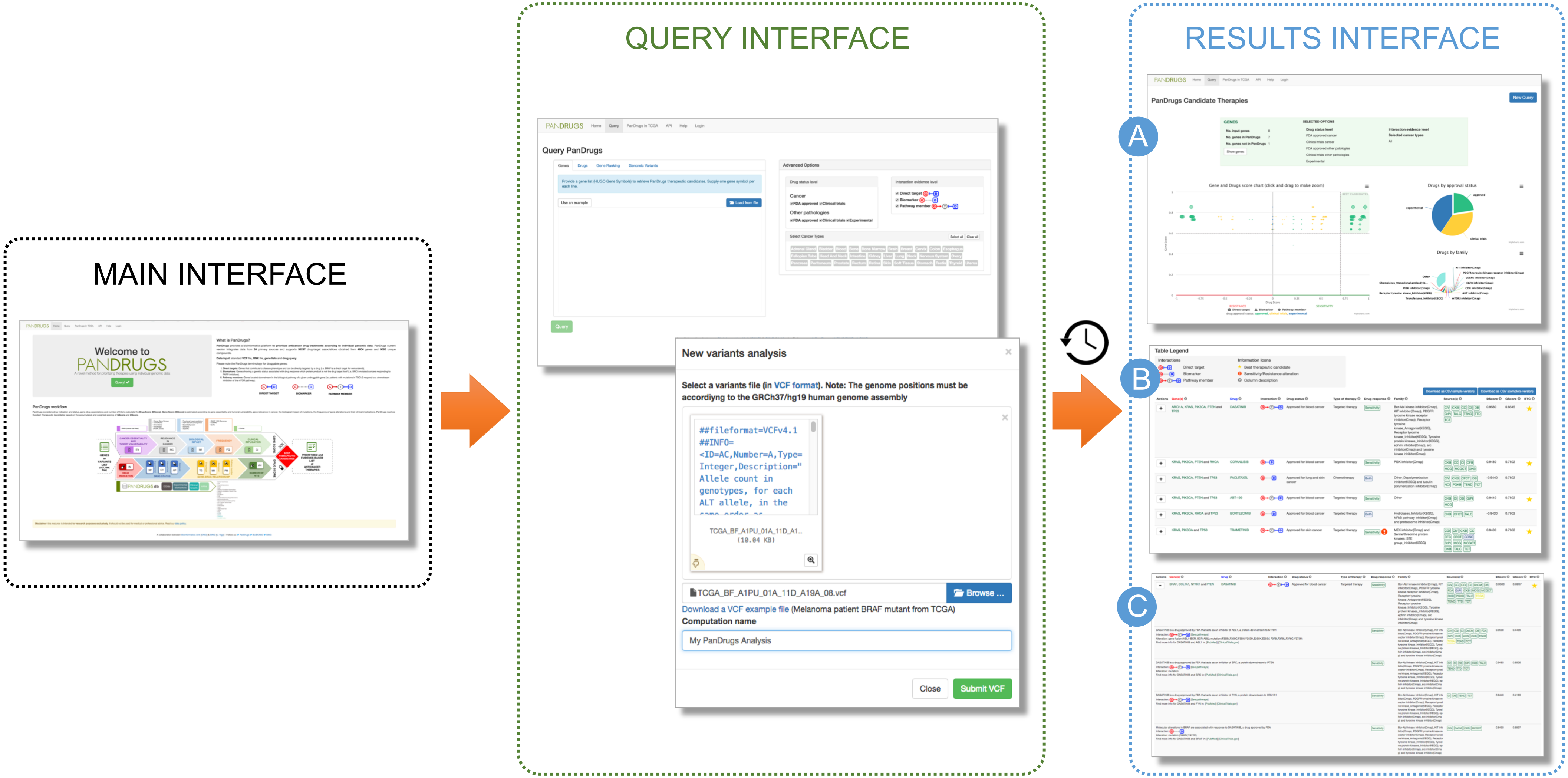

Supplementary Figure S4. PanDrugs workflow for standard VCF file containing variants from a BRAF-mutated melanoma patient from TCGA (TCGA-BF-A1PU-01A-11D-A19A-08). PanDrugs results interface includes: A) Score chart displaying the best candidates drugs proposed and B) summary table listing the treatments suggested. C) Summary table may be deployed to show detailed information for treatments (i.e. Dasatinib). Additional information about genes, annotation sources, drug characteristics and type of gene-drug interaction is displayed for each drug-gene association.

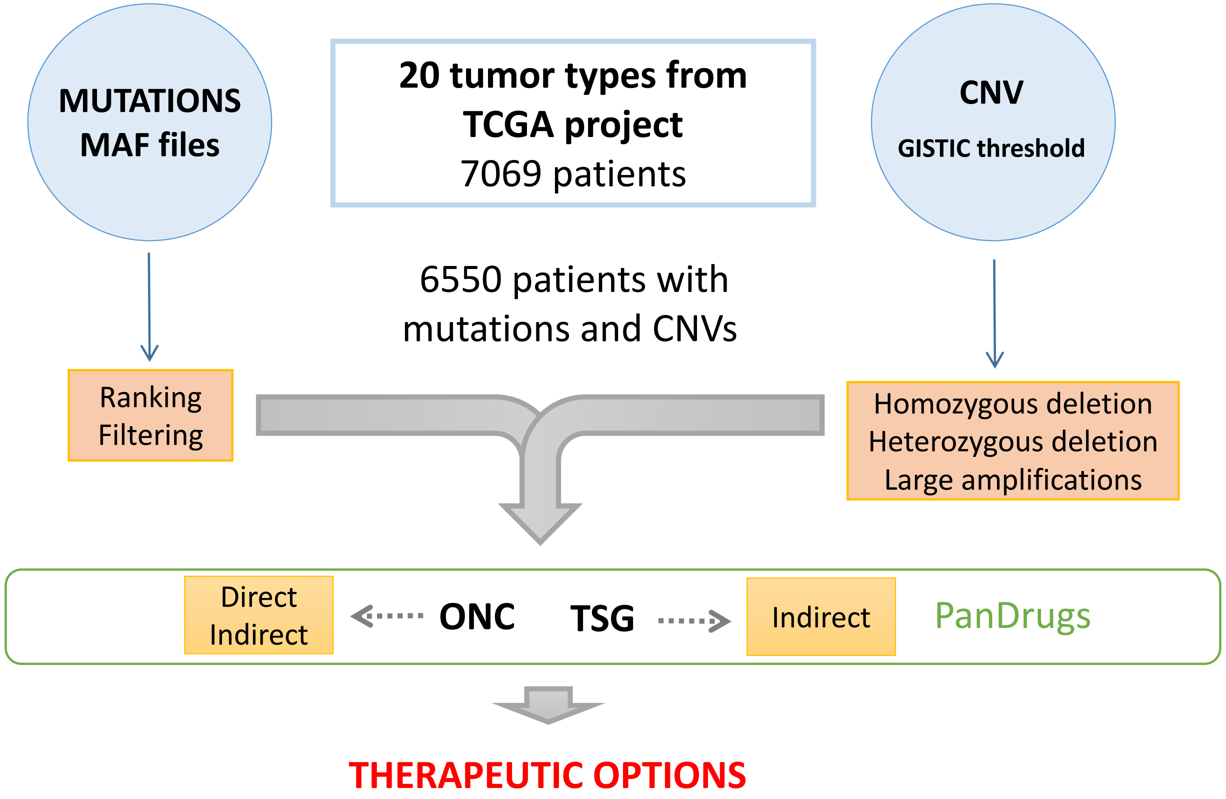

Supplementary Figure S5. Flow chart of the TCGA analysis. (ONC: Oncogenes; TSG: Tumor Supressor Genes.)

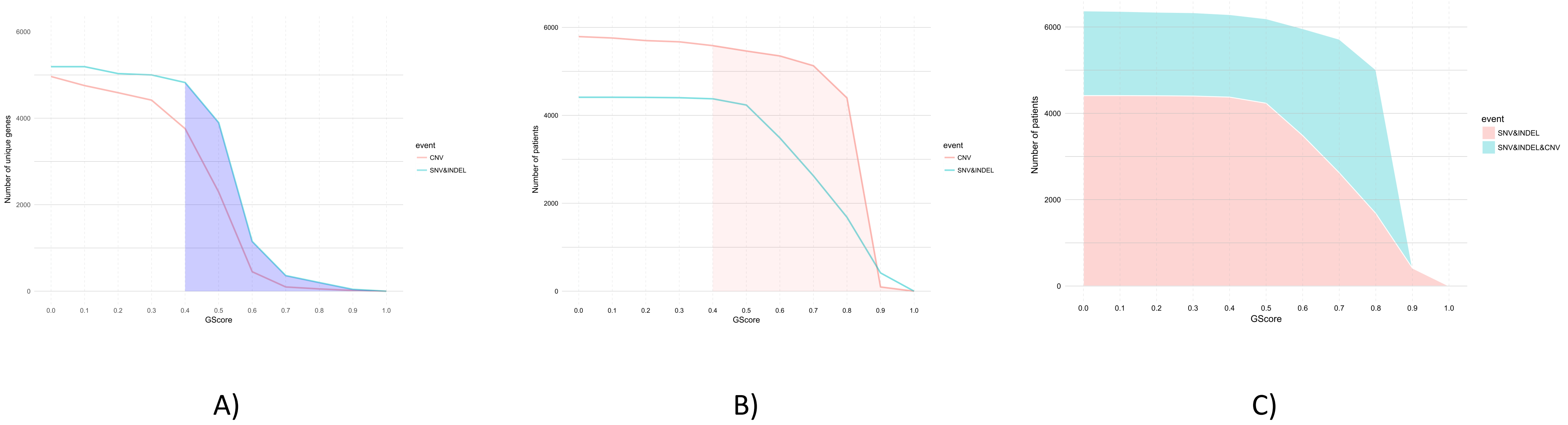

Supplementary Figure S6. A) Distribution of altered genes and their corresponding GScore value in TCGA data (SNVs, indels and CNVs). B) Distribution of patients and the corresponding accumulative GScore value in TCGA data (SNV, indels and CNVs). C) Distribution of patients and the corresponding accumulative GScore value in TCGA data (SNV+indels and SNV+indels+CNV).

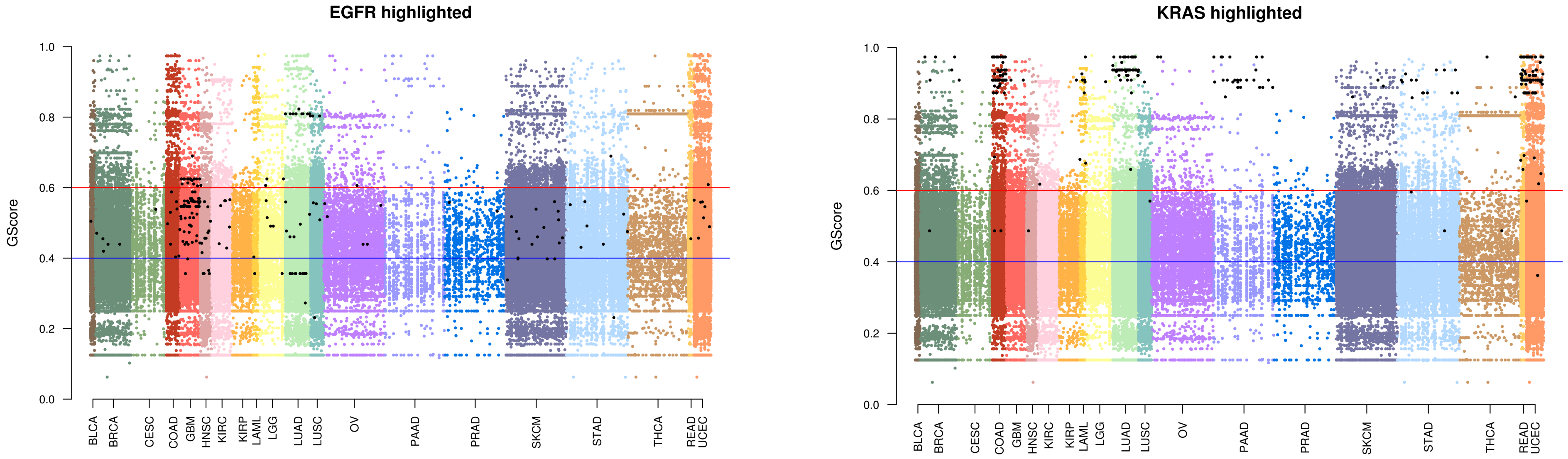

Supplementary Figure S7. Manhattan plots for GScore values across 20 TCGA tumor types. EGFR and KRAS GScores are highlighted in black.

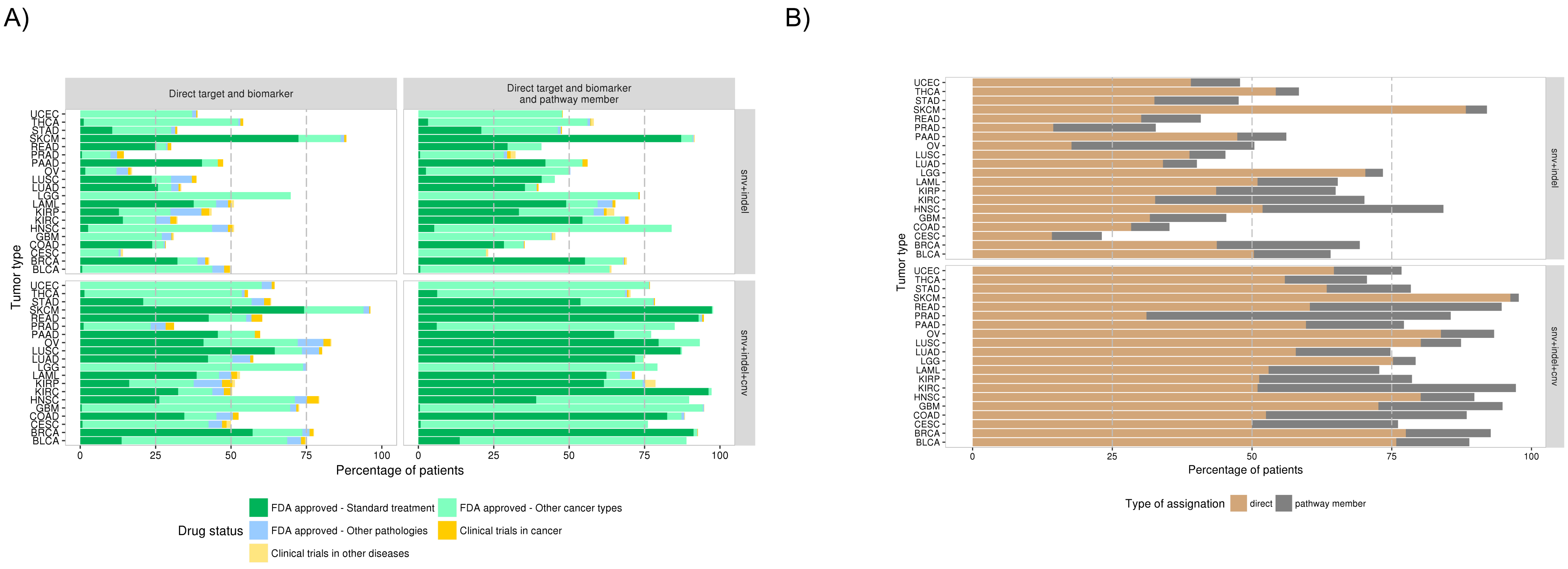

Supplementary Figure S8. A) TCGA patients under clinical trials or treated with approved drugs. Three different levels of evidence for these treatments are shown: (i) treatments that directly target the affected gene, (ii) treatments indicated to direct gene targets and biomarkers and, (iii) treatments indicated to direct gene targets, biomarkers and downstream pathway members. The bar-chart considers separately genes affected only by single nucleotide variants and indels (top panel) and genes affected by single nucleotide variants, indels and CNVs (bottom panel). B) TCGA patients treated with drugs that directly targets an altered gene (brown bars). Grey bars represent the increase in the number of TCGA patients treated when pathway members are also included. The bar-chart considers separately genes affected only by single nucleotide variants and indels (top panel) and genes affected by single nucleotide variants, indels and CNVs (bottom panel).

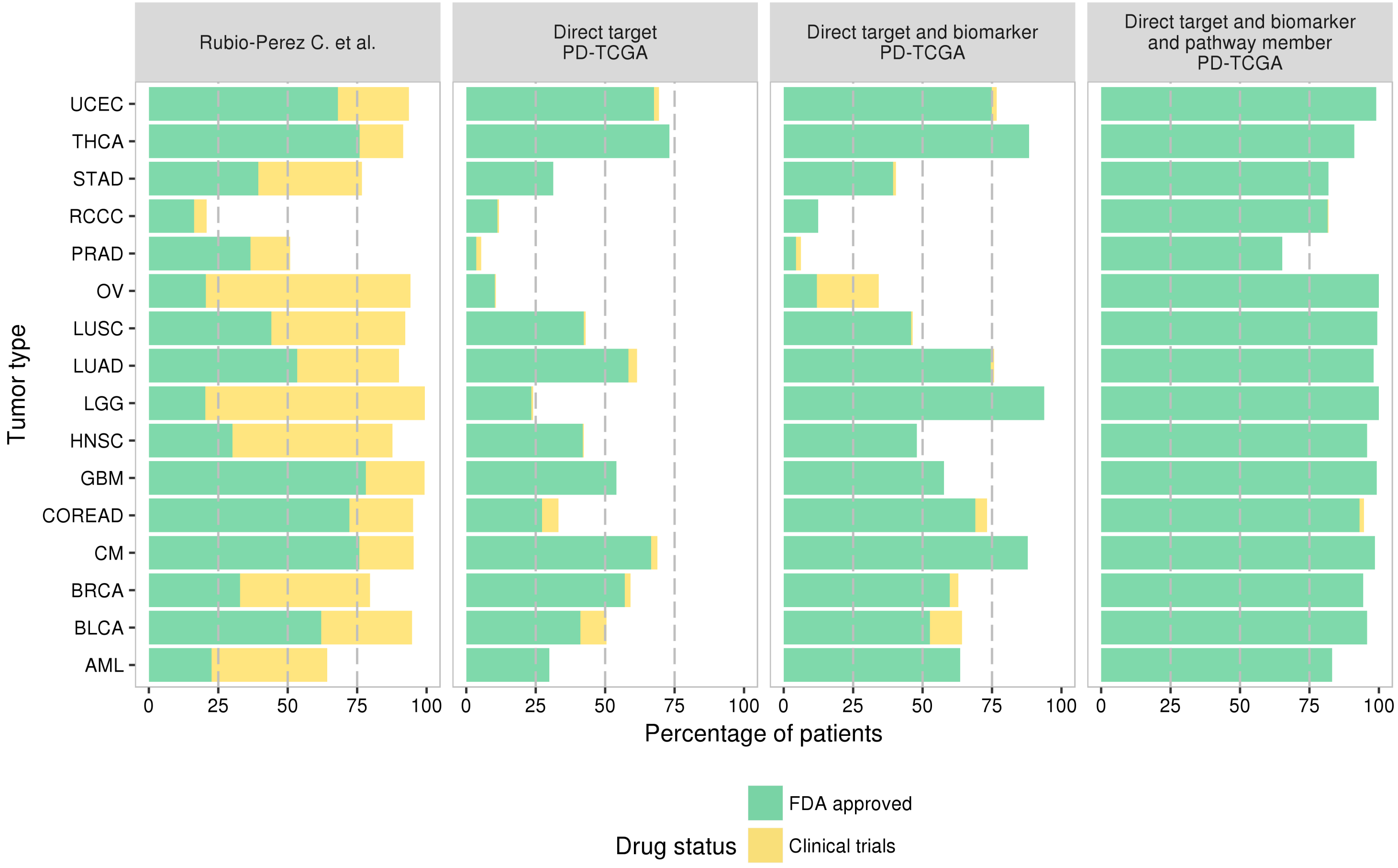

Supplementary Figure S9. Current in silico prescription methods based on the genomic analysis of known cancer genes may be enriched by PanDrugs pathway member approach. TCGA comparative analysis showed that PanDrugs path member approach clearly expands the number of cancer patients who can be potentially benefited with FDA approved treatments (green bars).

A)

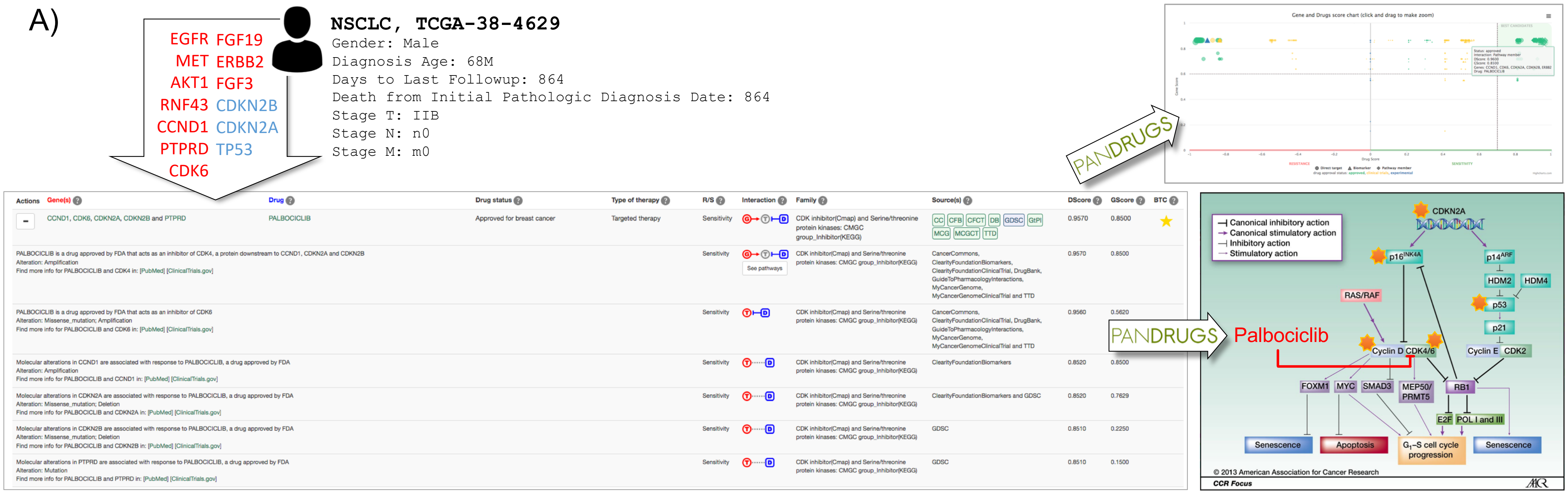

CDK4 pathway adapted from Sheppard KE and McArthur GA. Clin Cancer Res 2013; 19(19); 5320–8.

B)

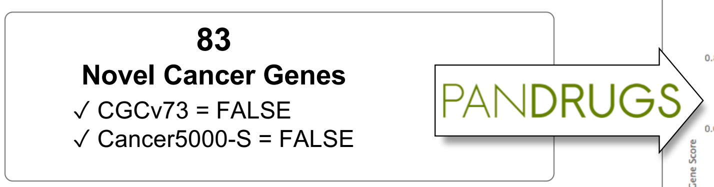

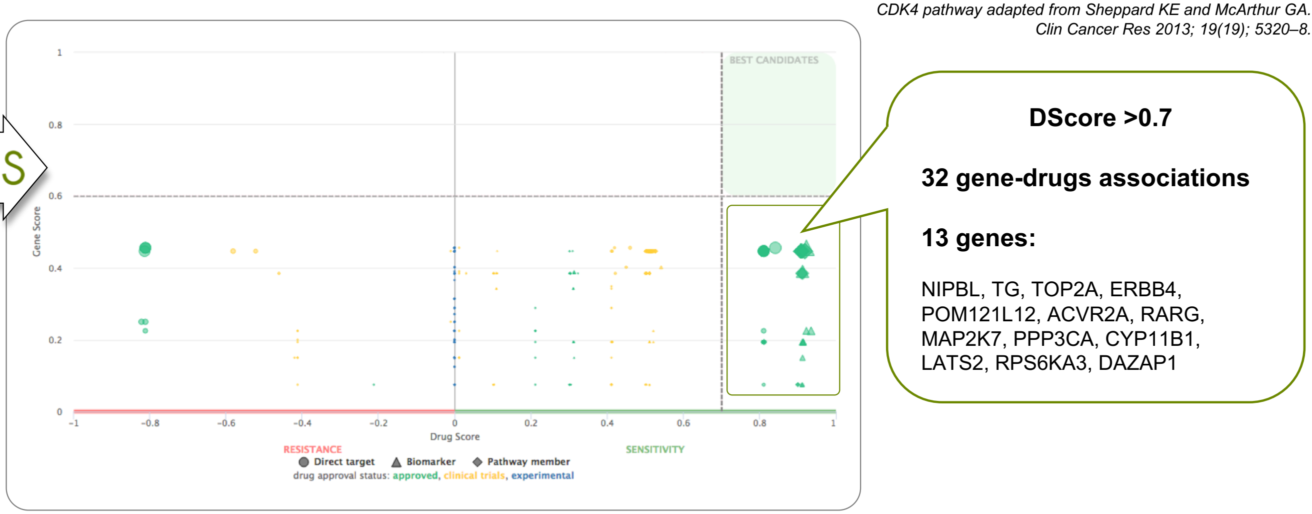

Supplementary Figure S10. (A) PanDrugs analysis for NSCLC patient (TCGA-38-4629). Oncogenes (red) and tumor supressors genes (blue) annotated in cBioportal were selected to make tool comparison viable. PanDrugs prescribes Palbociclib since (i) CDK6 is direct target, (ii) CCND1, CDKN2A and CDKN2B are biomarkers and, (iii) CDK4 is a downstream pathway member gene. (B) PanDrugs analysis for novel cancer genes proposed by Martincorena and colleagues. 13 genes showed DScore > 0.7 and were proposed as candidate targets. Low GScore in these genes is explained by the lack of clinical evidence and cancer annotations associated to them.

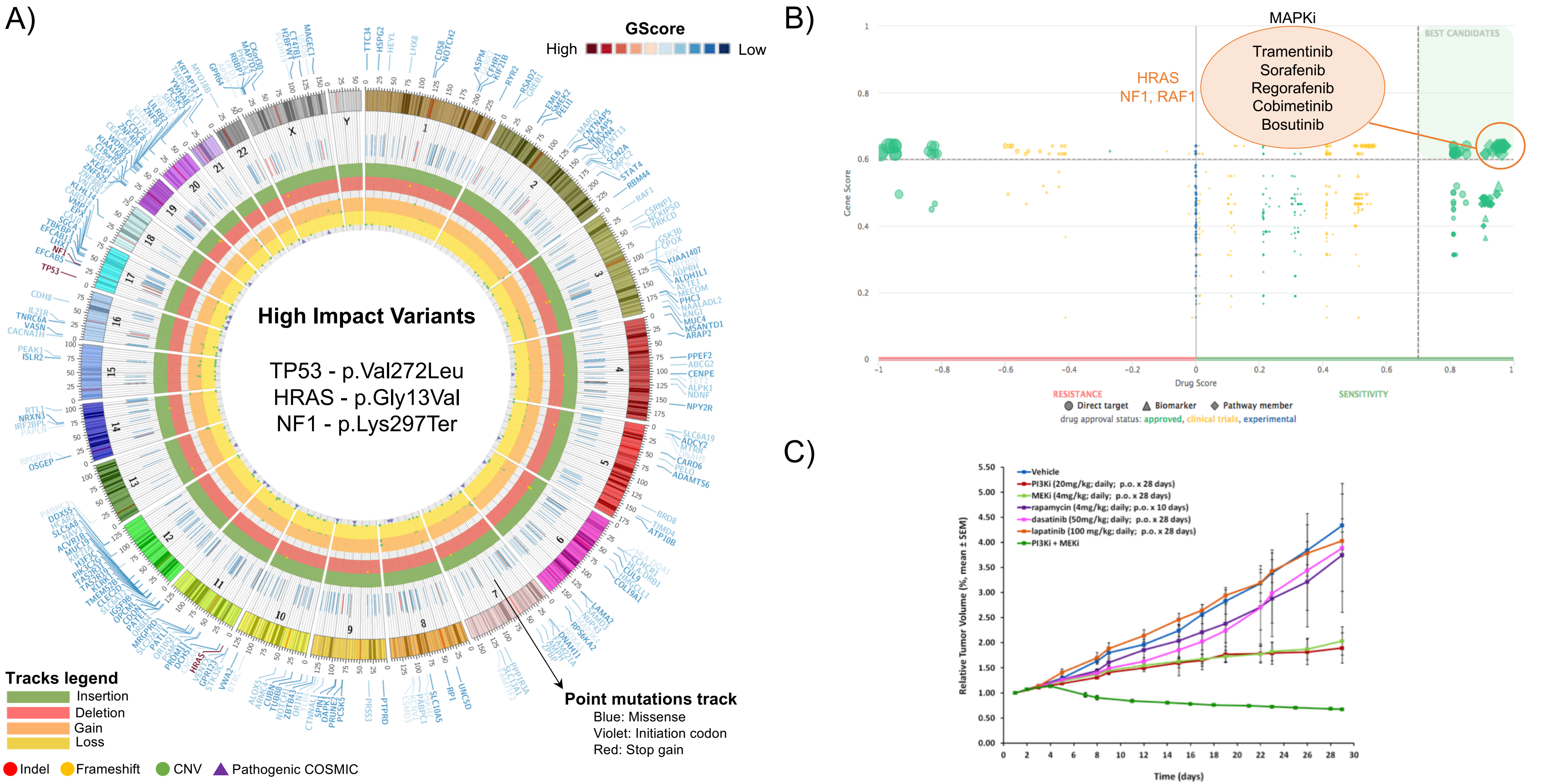

Supplementary Figure S11. PanDrugs has been employed to predict treatments in a PDX model of brain metastasis in advanced squamous cell lung carcinoma (Stage IV). A) Landscape of small genomic alterations detected in the PDX model. B) Drug assignations obtained from PanDrugs execution. C) Patient-derived xenograft tumor growth inhibition measure under drug efficacy test.

Source	Source provider	# initial records	# processed records	Direct Target /Biomarker	Sensitivity/Resistance	Alteration type	Expert curated
**CancerCommons**	DGIdb	104	104	yes			yes
**CGI**	DGIdb	309	309	yes	yes	yes	yes
**ChemblInteractions**	DGIdb	7695	7558				
**CIViC**	DGIdb	534	534		yes	yes	yes
**CKB**	DGIdb	1412	1403		yes	yes	yes
**ClearityFoundationBiomarkers**	DGIdb	148	148	yes	yes	yes	yes
**ClearityFoundationClinicalTrial**	DGIdb	178	175				yes
**DoCM**	DGIdb	72	72		yes	yes	yes
**DrugBank**	DGIdb	7805	7723	yes			
**FDA**	DGIdb	245	244	yes	yes	yes	yes
**GuideToPharmacologyInteractions**	DGIdb	7672	7613	yes			yes
**MyCancerGenome**	DGIdb	814	782	yes	yes		yes
**MyCancerGenomeClinicalTrial**	DGIdb	319	303		yes		yes
**NCI**	DGIdb	4298	4287	yes	yes	yes	yes
**OncoKB**	DGIdb	155	155			yes	yes
**PharmGKB**	DGIdb	1274	1245				
**TALC**	DGIdb	492	486	yes	yes		yes
**TdgClinicalTrial**	DGIdb	4155	4085	yes			yes
**TTD**	DGIdb	1829	2210	yes			
**TEND**	DGIdb	2233	1822	yes			yes
**moAb**	moAb	605	605	yes			yes
**TARGET-CGA**	TARGET	74	72	yes	yes	yes	yes
**CTRP**	CTRP	397270	13041		yes		
**GDSC**	GDSC	1323	1321		yes		
**Total**		441015	56297				

Supplementary Table 1. Gene-drug records in PanDrugsdb for each annotation source (redundant records are included). The table shows the number of initial records, the number of records after PanDrugsdb processing and the additional information provided.

Feature	Weight	Value	Score
**Essentiality Score**	40%	Computed Essentiality Score	[0 - 1]
**OncoScape Score**	30%	max{max{OncoScape score for oncogene in different tumor types}, max{OncoScape score for tumor suppressor gene in different tumor types}} normalized between 0 and 1	[0 - 1]
**Gene annotated in TumorPortal**	10%	Highly significantly mutated	1
		Significantly mutated	0.5
		Near significance	0.25
		No annotation	0
**Gene annotated in Cancer Gen Census (COSMIC)**	10%	Yes	1
		No	0
**Driver Gene**	10%	High confidence driver	1
		Candidate driver	0.5
		No annotation	0

Supplementary Table 2. Features involved and their corresponding weights in PanDrugs GScore calculation for non-ranked lists of genes.

Feature	Value	Score addition ONC	Score addition TSG
**Score prediction by PolyPhen**	> 0.435	+ 0.125/3	+ 0.125/3
**Score prediction by Sift**	<= 0.05	+ 0.125/3	+ 0.125/3
**Score prediction by CONDEL**	> 0.468	+ 0.125/3	+ 0.125/3
**COSMIC**	Pathogenic by FATHMM prediction	+ 0.125/3	+ 0.03125
**Frequency of mutation in COSMIC**	>= 100	+ 0.125/3	
	< 100	+ (0.125 / 3) * (log(mutation frequency) / log(maximum mutation frequency))	
**Frequency of gene in COSMIC**	>= 100	+ 0.125/3	+ 0.03125
	< 100	+ (0.125 / 3) * (log(gene frequency) / log(maximum gene frequency))	+ 0.03125 * (log(gene frequency) / log(maximum gene frequency))
**VEP consequence**	stop gain<br>frameshift<br>missense<br>inframe insertion<br>inframe deletion	+ 0.125	+ 0.125
**GMAF**	< 1	+ 0.125/2	+ 0.125/2
**EXAC**	< 1	+ 0.125/2	+ 0.125/2
**DOMAINS**	Listed as relevant in cancer[1] or previous last protein domain (in stop-gained or frameshift)	+ 0.125	+ 0.125
	Within a domain in other circumstances	+ 0.125/2	+ 0.125/2
**CLINVAR**	Pathogenic with zygosity data	+ 0.125	+ 0.125
	Pathogenic without zygosity data	+ 0.250	+ 0.3125
**ZYGOSITY (when available)**	Homozygous	+ 0.125	+ 0.1875
**ESSENTIALITY SCORE**		+ 0.125 * ES	+ 0.125 * ES

[1] *Yang F, et al. Proteindomain-level landscape of cancer-type-specific somatic mutations. PLoS Comput Biol. 2015 Mar 20;11(3):e1004147.*

Supplementary Table 3. PanDrugs GScore weight assignation for lists of gene variants (VCF files). For each variant PanDrugs calculates a variant score (VScore). Highest VScore for each gene is selected as the GScore. (ONC: Oncogene; TSG: Tumor suppressor gene.)

Tumor type	TCGA Code	Mutations (Synapse IDs)	CNV (Synapse IDs)
**Bladder Urothelial Carcinoma**	BLCA	syn1729383	syn1687592
**Breast invasive carcinoma**	BRCA	syn1729383	syn395566
**Cervical squamous cell carcinoma and endocervical adenocarcinoma**	CESC	syn1729383	syn1687594
**Colon adenocarcinoma**	COAD	syn1729383	syn1687596
**Rectum adenocarcinoma**	READ	syn1729383	syn1687628
**Glioblastoma multiforme**	GBM	syn1729383	syn1687604
**Head and Neck squamous cell carcinoma**	HNSC	syn1729383	syn1687600
**Kidney renal clear cell carcinoma**	KIRC	syn1729383	syn1687602
**Kidney renal papillary cell carcinoma**	KIRP	syn1729383	syn1687614
**Acute Myeloid Leukemia**	LAML	syn1729383	syn1714787
**Brain Lower Grade Glioma**	LGG	syn1729383	syn1687616
**Lung adenocarcinoma**	LUAD	syn1729383	syn1687610
**Lung squamous cell carcinoma**	LUSC	syn1729383	syn1687612
**Ovarian serous cystadenocarcinoma**	OV	syn1729383	syn1687638
**Pancreatic adenocarcinoma**	PAAD	syn1729383	syn1687626
**Prostate adenocarcinoma**	PRAD	syn1729383	syn1687640
**Skin Cutaneous Melanoma**	SKCM	syn1729383	syn1687618
**Stomach adenocarcinoma**	STAD	syn1729383	syn1687622
**Thyroid carcinoma**	THCA	syn1729383	syn1687634
**Uterine Corpus Endometrioid Carcinoma**	UCEC	syn1729383	syn1687636

Supplementary Table 4. File sources for TCGA data employed by PanDrugs analysis.

Resource	Version
**ensembl**	version 90
**COSMIC**	Release 84
**Pfam**	31.0
**UniProt**	2018_02
**InterPro**	66.0
**Clinvar**	2018_02
**APPRIS**	gencode19/ensembl74

Supplementary Table 5. Database versions employed in the TCGA analysis.

Tumor type	Initial number of patients	Alteration type	Number of patients (SNV&Indels/CNV/Both)
**BLCA**	153	MUT/CNV/BOTH	99/135/96
**BRCA**	934	MUT/CNV/BOTH	771/866/752
**CESC**	134	MUT/CNV/BOTH	39/102/36
**COAD**	423	MUT/CNV/BOTH	155/413/153
**READ**	169	MUT/CNV/BOTH	69/162/68
**GBM**	599	MUT/CNV/BOTH	291/563/281
**HNSC**	343	MUT/CNV/BOTH	306/306/302
**KIRC**	502	MUT/CNV/BOTH	417/493/415
**KIRP**	117	MUT/CNV/BOTH	100/103/100
**LAML**	202	MUT/CNV/BOTH	196/194/190
**LGG**	222	MUT/CNV/BOTH	170/180/169
**LUAD**	543	MUT/CNV/BOTH	230/356/172
**LUSC**	389	MUT/CNV/BOTH	178/343/178
**OV**	599	MUT/CNV/BOTH	316/559/311
**PAAD**	57	MUT/CNV/BOTH	34/48/34
**PRAD**	180	MUT/CNV/BOTH	83/171/82
**SKCM**	264	MUT/CNV/BOTH	253/236/225
**STAD**	292	MUT/CNV/BOTH	151/237/115
**THCA**	435	MUT/CNV/BOTH	323/401/318
**UCEC**	512	MUT/CNV/BOTH	248/492/242
**Total**	7069		4429/6360/4239

Supplementary Table 6. Patients and genomic alterations considered in the TCGA data analysis sorted by tumor type.

Supplementary Table 7. Most frequently altered genes suggested for treatment by PanDrugs. The table shows the top-5 genes for each TCGA tumor type considering snv (point mutations and indels) and for CNVs separately.

Tumor	Gene	# cases	Event	Driver gene
**BLCA**	TP53	90	cnv	yes
**BLCA**	CDKN2A	81	cnv	yes
**BLCA**	CDKN2B	81	cnv	no
**BLCA**	LPL	80	cnv	no
**BLCA**	PTK2B	80	cnv	no
**BLCA**	TP53	50	snv	yes
**BLCA**	SYNE1	22	snv	yes
**BLCA**	PIK3CA	18	snv	yes
**BLCA**	RB1	14	snv	yes
**BLCA**	CDKN1A	13	snv	yes
**BRCA**	TP53	593	cnv	yes
**BRCA**	MC1R	571	cnv	no
**BRCA**	CYBA	569	cnv	no
**BRCA**	PLCG2	563	cnv	no
**BRCA**	PARD6A	550	cnv	no
**BRCA**	PIK3CA	261	snv	yes
**BRCA**	TP53	256	snv	yes
**BRCA**	CDH1	57	snv	yes
**BRCA**	MAP3K1	57	snv	yes
**BRCA**	MAP2K4	32	snv	yes
**CESC**	ETS1	60	cnv	no
**CESC**	TP53AIP1	60	cnv	no
**CESC**	ARHGEF12	59	cnv	yes
**CESC**	CHEK1	59	cnv	no
**CESC**	THY1	59	cnv	no
**CESC**	PIK3CA	9	snv	yes
**CESC**	NFE2L2	6	snv	yes
**CESC**	MYH9	5	snv	yes
**CESC**	CREBBP	4	snv	yes
**CESC**	SYNE1	4	snv	yes
**COAD**	SMAD4	270	cnv	yes
**COAD**	DCC	269	cnv	yes
**COAD**	MALT1	264	cnv	yes
**COAD**	TNFRSF11A	260	cnv	no
**COAD**	NFATC1	259	cnv	no
**COAD**	TP53	76	snv	yes
**COAD**	KRAS	59	snv	yes
**COAD**	SYNE1	38	snv	yes
**COAD**	PIK3CA	32	snv	yes
**COAD**	LRP2	29	snv	no
**GBM**	PTEN	505	cnv	yes
**GBM**	DOCK1	501	cnv	no
**GBM**	PLCE1	500	cnv	no
**GBM**	BLNK	499	cnv	no
**GBM**	CASP7	497	cnv	no
**GBM**	PTEN	90	snv	yes
**GBM**	TP53	84	snv	yes
**GBM**	EGFR	77	snv	yes

**GBM**	NF1	32	snv	yes
**GBM**	PIK3CA	32	snv	yes
**HNSC**	PRKCD	234	cnv	yes
**HNSC**	RHOA	234	cnv	yes
**HNSC**	TLR9	234	cnv	no
**HNSC**	WNT5A	234	cnv	no
**HNSC**	APPL1	233	cnv	no
**HNSC**	TP53	215	snv	yes
**HNSC**	CDKN2A	66	snv	yes
**HNSC**	PIK3CA	64	snv	yes
**HNSC**	SYNE1	56	snv	yes
**HNSC**	FAM135B	30	snv	yes
**KIRC**	VHL	445	cnv	yes
**KIRC**	CDC25A	437	cnv	no
**KIRC**	CTNNB1	435	cnv	yes
**KIRC**	CXCR6	435	cnv	no
**KIRC**	MYD88	435	cnv	yes
**KIRC**	VHL	218	snv	yes
**KIRC**	MTOR	25	snv	yes
**KIRC**	PTEN	18	snv	yes
**KIRC**	SYNE1	16	snv	yes
**KIRC**	FBN2	12	snv	yes
**KIRP**	MAPK12	30	cnv	no
**KIRP**	MAPK8IP2	29	cnv	no
**KIRP**	ADORA2A	28	cnv	yes
**KIRP**	BCR	28	cnv	yes
**KIRP**	CSNK1E	28	cnv	no
**KIRP**	MET	8	snv	yes
**KIRP**	SYNE1	6	snv	yes
**KIRP**	BRAF	4	snv	yes
**KIRP**	KAT6A	4	snv	yes
**KIRP**	LRP2	4	snv	no
**LAML**	FLT3	54	cnv	yes
**LAML**	CDK5	24	cnv	no
**LAML**	CUL1	23	cnv	yes
**LAML**	IRF5	23	cnv	no
**LAML**	LEP	23	cnv	no
**LAML**	FLT3	53	snv	yes
**LAML**	IDH2	20	snv	yes
**LAML**	IDH1	19	snv	yes
**LAML**	RUNX1	18	snv	yes
**LAML**	TP53	16	snv	yes
**LGG**	IDH1	131	cnv	yes
**LGG**	GP6	101	cnv	no
**LGG**	KIR2DL1	100	cnv	no
**LGG**	KIR3DL1	100	cnv	no
**LGG**	KIR3DL2	100	cnv	no
**LGG**	IDH1	131	snv	yes
**LGG**	TP53	88	snv	yes
**LGG**	PIK3CA	15	snv	yes
**LGG**	NF1	11	snv	yes
**LGG**	EGFR	8	snv	yes
**LUAD**	TP53	265	cnv	yes

**LUAD**	CDKN2A	217	cnv	yes
**LUAD**	CDKN2B	206	cnv	no
**LUAD**	NLRP1	206	cnv	no
**LUAD**	SERPINF1	206	cnv	no
**LUAD**	TP53	122	snv	yes
**LUAD**	KRAS	60	snv	yes
**LUAD**	FBN2	40	snv	yes
**LUAD**	KEAP1	40	snv	yes
**LUAD**	SYNE1	39	snv	yes
**LUSC**	RASSF1	297	cnv	no
**LUSC**	TLR9	297	cnv	no
**LUSC**	WNT5A	297	cnv	no
**LUSC**	APPL1	296	cnv	no
**LUSC**	PRKCD	295	cnv	yes
**LUSC**	TP53	145	snv	yes
**LUSC**	SYNE1	52	snv	yes
**LUSC**	FAM135B	33	snv	yes
**LUSC**	LRP2	32	snv	no
**LUSC**	SI	30	snv	no
**OV**	APC2	495	cnv	no
**OV**	S1PR4	492	cnv	no
**OV**	GNA11	491	cnv	yes
**OV**	MKNK2	491	cnv	no
**OV**	STK11	491	cnv	yes
**OV**	TP53	301	snv	yes
**OV**	CSMD3	18	snv	yes
**OV**	LRP2	15	snv	no
**OV**	NF1	12	snv	yes
**OV**	SYNE1	9	snv	yes
**PAAD**	SMAD4	31	cnv	yes
**PAAD**	TP53	31	cnv	yes
**PAAD**	CDKN2A	30	cnv	yes
**PAAD**	DCC	29	cnv	yes
**PAAD**	PIAS2	28	cnv	no
**PAAD**	KRAS	24	snv	yes
**PAAD**	TP53	22	snv	yes
**PAAD**	SMAD4	8	snv	yes
**PAAD**	CDKN2A	6	snv	yes
**PAAD**	MYH9	6	snv	yes
**PRAD**	GNRH1	97	cnv	no
**PRAD**	LPL	97	cnv	no
**PRAD**	PTK2B	90	cnv	no
**PRAD**	GATA4	87	cnv	no
**PRAD**	HTR2A	74	cnv	no
**PRAD**	SYNE1	5	snv	yes
**PRAD**	TP53	5	snv	yes
**PRAD**	HSPG2	4	snv	no
**PRAD**	BCL6	3	snv	yes
**PRAD**	CTNNB1	3	snv	yes
**READ**	DCC	143	cnv	yes
**READ**	SMAD4	143	cnv	yes
**READ**	MALT1	142	cnv	yes
**READ**	TNFRSF11A	142	cnv	no

**READ**	PMAIP1	141	cnv	no
**READ**	TP53	45	snv	yes
**READ**	KRAS	38	snv	yes
**READ**	PIK3CA	13	snv	yes
**READ**	SYNE1	11	snv	yes
**READ**	SMAD4	8	snv	yes
**SKCM**	CDKN2A	187	cnv	yes
**SKCM**	CDKN2B	182	cnv	no
**SKCM**	CER1	171	cnv	no
**SKCM**	IFNA1	171	cnv	no
**SKCM**	IFNB1	171	cnv	no
**SKCM**	BRAF	132	snv	yes
**SKCM**	NRAS	70	snv	yes
**SKCM**	SYNE1	61	snv	yes
**SKCM**	PREX2	59	snv	yes
**SKCM**	DCC	56	snv	yes
**STAD**	TP53	120	cnv	yes
**STAD**	DCC	107	cnv	yes
**STAD**	SMAD4	101	cnv	yes
**STAD**	TNFRSF11A	101	cnv	no
**STAD**	MALT1	100	cnv	yes
**STAD**	TP53	69	snv	yes
**STAD**	SYNE1	48	snv	yes
**STAD**	PREX2	28	snv	yes
**STAD**	PIK3CA	25	snv	yes
**STAD**	LRP2	24	snv	no
**THCA**	BRAF	187	cnv	yes
**THCA**	MAPK8IP2	71	cnv	no
**THCA**	CSF2RB	70	cnv	no
**THCA**	MAPK12	70	cnv	no
**THCA**	PPARA	70	cnv	no
**THCA**	BRAF	183	snv	yes
**THCA**	NRAS	26	snv	yes
**THCA**	HRAS	12	snv	yes
**THCA**	MT-ND5	9	snv	no
**THCA**	LRP1	6	snv	no
**UCEC**	PTEN	230	cnv	yes
**UCEC**	PLCG2	163	cnv	no
**UCEC**	PHLPP2	160	cnv	no
**UCEC**	PIK3CA	160	cnv	yes
**UCEC**	CDH1	157	cnv	yes
**UCEC**	PTEN	161	snv	yes
**UCEC**	PIK3CA	132	snv	yes
**UCEC**	CTNNB1	74	snv	yes
**UCEC**	TP53	69	snv	yes
**UCEC**	KRAS	53	snv	yes

Supplementary Table 8. Summary of 46 deleterious variants detected in SCLC patient enrolled for personalized medicine protocol.

chr	Position	Mutation	Consequence	Gene Symbol	Amino Acid Change	dbSNP	Lung Squamous Cell Carcinoma (TCGA) % Cases Altered	COSMIC ID	COSMIC Gene frequency
1	215960153	A/C	missense variant	USH2A	C3416G		36.50%	130095	649 / 606592
1	120612003	GG/-	frameshift variant, feature truncation	NOTCH2	6		9%		
3	119634983	G/C	missense variant	GSK3B	I172M		7.30%		18 / 606592
3	171417616	C/A	missense variant	PLD1	W382C		38.20%		73 / 606592
3	48723095	G/A	missense variant	NCKIPSD	P49L		1.70%		12 / 606592
3	53220704	C/T	missense variant	PRKCD	R449C		1.70%		18 / 606592
3	12626663	C/A	missense variant	RAF1	M562I		0.60%		32 / 606592
3	127800173	C/T	missense variant	RUVBL1	E431K		7.30%	172449	28 / 606592
3	46414969	-/GCTCTCAT	frameshift variant, feature elongation	CCR5	192		1.70%		
4	113356408	A/G	missense variant	ALPK1	R969G		1.70%		61 / 606592
4	106156522	ATGCTTTCTGAAAGGCCTCAGAATA/-	frameshift variant, feature truncation	TET2	475-483		2.80%		
5	156381503	C/A	missense variant	TIMD4	G108V		4.50%	402725	49 / 606592
5	140052407	G/A	missense variant	DND1	P76L	rs72800920	0.60%	1130919	6 / 606592
5	37044488	A/C	missense variant	NIPBL	I2050L		13.50%		134 / 606592
5	13864613	C/A	stop gained	DNAH5	E1497*		26.40%	232179	
6	107008770	G/T	missense variant	AIM1	R1575L		5.60%		67 / 606592
6	161508864	C/G	missense variant	MAP3K4	L901V		3.90%		81 / 606592
6	166827293	G/A	missense variant	RPS6KA2	H600Y		2.80%		43 / 606592
7	113558424	G/T	missense variant	PPP1R3A	R210S	rs141223649	8.40%		134 / 606592
8	88885088	C/T	missense variant	DCAF4L2	R371H		5.60%	1102264	97 / 606592
8	101719121	G/A	missense variant	PABPC1	R436C	rs79986761	1.10%	748078	43 / 606592
8	56922484	G/T	missense variant	LYN	V431L		3.90%		23 / 606592
8	87076765	A/T	missense variant	PSKH2	M94K		3.40%		46 / 606592
8	113516090	G/C	stop gained	CSMD3	S1671*		44.40%	603817	
9	90114003	T/A	missense variant	DAPK1	F4Y		1.70%		79 / 606592
9	8317878	G/A	missense variant	PTPRD	T1890M	rs151311972	12.40%		184 / 606592
9	120476517	A/T	missense variant	TLR4	Q640L		5.60%		117 / 606592

10	134059432	C/A	missense variant	STK32C	R36L		1.10%		20 / 606592
10	103221746	-/AG	frameshift variant, feature elongation	BTRC	19		1.70%		
11	534285	C/A	missense variant	HRAS	G13V	rs104894226	3.40%	489	21 / 606592
11	123777861	T/A	stop gained	OR8D4	C241*	rs61748875	2.80%	147344	
12	52369253	A/G	missense variant	ACVR1B	D47G		1.70%		41 / 606592
12	78513222	T/A	missense variant	NAV3	S154R		20.20%		261 / 606592
12	18439806	T/A	missense variant	PIK3C2G	V235E		5.10%		63 / 606592
12	56488226	G/T	missense variant	ERBB3	G523V		2.20%		2 / 606592
12	53605639	T/C	missense variant	RARG	R324G		2.20%		20 / 606592
14	33291955	T/A	missense variant	AKAP6	S1646T		9%		140 / 606592
14	21785937	C/T	stop gained	RPGRIP1	Q54*		1.10%	552574	
16	74502926	C/A	missense variant	GLG1	R774L		2.80%		61 / 606592
17	7577124	C/A	missense variant	TP53	V272L	rs121912657	90.40%	133678	1365 / 606592
17	78172215	G/T	missense variant	CARD14	R322L		1.70%		25 / 606592
17	29546102	C/G	stop gained	NF1	S202*		11.80%	96520	
17	29527440	A/T	stop gained, splice region variant	NF1	K297*		11.80%	41809	
19	10602314	C/T	missense variant	KEAP1	D422N		14%	710198	100 / 606592
19	11129683	T/A	missense variant	SMARCA4	V830E		6.20%		136 / 606592
22	26219574	T/A	missense variant	MYO18B	L875H		11.20%		165 / 606592
